# Variation in Target Attainment of Beta‐Lactam Antibiotic Dosing Between International Pediatric Formularies

**DOI:** 10.1002/cpt.2180

**Published:** 2021-02-28

**Authors:** Silke Gastine, Yingfen Hsia, Michelle Clements, Charlotte I.S. Barker, Julia Bielicki, Christine Hartmann, Mike Sharland, Joseph F. Standing

**Affiliations:** ^1^ Infection, Immunity and Inflammation Research and Teaching Department UCL Great Ormond Street Institute of Child Health University College London London UK; ^2^ School of Pharmacy Queen’s University Belfast Belfast UK; ^3^ MRC Clinical Trials Unit University College London London UK; ^4^ Department of Medical & Molecular Genetics King’s College London London UK; ^5^ Paediatric Infectious Diseases Research Group Institute for Infection and Immunity St George’s University of London London UK; ^6^ Paediatric Pharmacology Group University of Basel Children’s Hospital Basel Switzerland; ^7^ Department of Pharmacy Great Ormond Street Hospital for Children London UK

## Abstract

As antimicrobial susceptibility of common bacterial pathogens decreases, ensuring optimal dosing may preserve the use of older antibiotics in order to limit the spread of resistance to newer agents. Beta‐lactams represent the most widely prescribed antibiotic class, yet most were licensed prior to legislation changes mandating their study in children. As a result, significant heterogeneity persists in the pediatric doses used globally, along with quality of evidence used to inform dosing. This review summarizes dosing recommendations from the major pediatric reference sources and tries to answer the questions: Does beta‐lactam dose heterogeneity matter? Does it impact pharmacodynamic target attainment? For three important severe clinical infections—pneumonia, sepsis, and meningitis—pharmacokinetic models were identified for common for beta‐lactam antibiotics. Real‐world demographics were derived from three multicenter point prevalence surveys. Simulation results were compared with minimum inhibitory concentration distributions to inform appropriateness of recommended doses in targeted and empiric treatment. While cephalosporin dosing regimens are largely adequate for target attainment, they also pose the most risk of neurotoxicity. Our review highlights aminopenicillin, piperacillin, and meropenem doses as potentially requiring review/optimization in order to preserve the use of these agents in future.

In a 2020 report on child mortality led by the United Nations Children’s Fund (UNICEF) and the World Health Organization (WHO),^1^ infectious diseases were found to be the leading cause of death in children under the age of 5 years and are estimated to cause over half of deaths in this age group.^2^ Bacterial infections, increasingly involving multidrug resistant organisms, are responsible for a significant proportion of these deaths, with pneumonia accounting for 15%, sepsis 7%, and meningitis 2%, respectively.^1^ In many cases these infectious diseases are preventable and need to be targeted by both policies aimed at prevention and also optimal treatment.

One barrier to optimal treatment is the rise of antimicrobial resistance (AMR), particularly among gram‐negative organisms. Over the last 20 years in Malawi, bloodstream isolates have changed from mainly sensitive to mainly resistant to first‐line antibiotics (gentamicin, ampicillin, and cefotaxime), with resistance in *Klebsiella species* now over 90%.^3^ This concerning trend is seen throughout other low‐income and middle‐income countries,^2^ and may mean that previous gains in reductions in mortality may be impacted by infections caused by multidrug resistant organisms.^4^ In an effort to reduce the spread of AMR, the WHO has categorized the Essential Medicines List (EML) agents into "Access," "Watch," and "Reserve" (AWaRe) antibiotics,^5^ to manage global antibiotic usage. This seeks to limit the use of “Watch” and “Reserve” agents where possible. *In vitro* there is evidence to suggest that low antibiotic exposure can select for or induce resistance, whereas when concentrations are higher resistance is less likely to appear.^6^ While clinical dosing guidelines should primarily recommend “Access” and “Watch” agents, a key aspect of preserving their efficacy is therefore to ensure dosing is optimized.

Historically, there were limited antimicrobial studies in children, but in 2003 the United States introduced the Best Pharmaceuticals for Children Act and the Pediatric Research Equity Act to ensure all new agents had to be studied;^7^ regulators in other territories followed suit shortly thereafter. This meant that new antimicrobial agents were formally studied—although those studies often lag significantly behind adult development.^7^ Since most antimicrobials in the WHO EML “Access” and “Watch” groups were licensed before 2003, inconsistent dosing guidelines are common. By far the largest proportion of “Access” and “Watch” agents are the beta‐lactams, which were licensed prior to these legislative changes and therefore variably studied in children.

Recent large global antimicrobial point prevalence surveys have confirmed that wide variability in antimicrobial dosing in children persists in clinical practice.^8^ Differences include the prescribed doses, frequencies, and in some cases the route of administration. Information on optimal dosing across different pediatric age groups is still lacking for many antibiotics and has often been omitted from drug labeling information. Formularies established by national and international expert groups are one of the main resources for pediatric drug dosing guidelines used in clinical care. Many of these have recently switched to digital platforms in order to increase their instant availability and contemporary information.^9^ Nevertheless, most formularies do not include references that informed the stated dosing recommendations. It is currently unclear whether the heterogeneity in international dosing guidelines is affecting antibiotic efficacy and safety in children, and if so whether certain dosing guidelines should be preferred over others.

For treatment to be successful, it is commonly recognized that attaining concentrations above a certain critical target is required.^10^ The usual pharmacodynamic marker that guides antibiotic efficacy is the minimum inhibitory concentration (MIC). Based on *in vitro* studies, the optimal pharmacokinetic target in relation to an organism’s MIC has been defined for each antibiotic: fC_max_/MIC, ratio of maximal free drug concentration to MIC; fAUC/MIC, ratio of the area under the free drug concentration‐time curve to MIC; fT > MIC, fraction of time of the free drug concentration being above the MIC, with the latter usually used for beta‐lactam antibiotics.^11^ However, to define a drug’s therapeutic window, an assessment of likely efficacy needs to be accompanied by an assessment of the proposed dose’s potential to cause adverse drug reactions. For some antibiotics with narrow therapeutic windows such as nephrotoxicity associated with aminoglycosides or glycopeptides, these toxicity cutoffs are well defined. For the beta‐lactams, toxicity thresholds are less well defined, but increasing evidence points towards neurotoxicity possibly becoming dose‐limiting, especially when new dosing regimens such as continuous infusion dosing are gaining in popularity.^12^

Focusing on three clinically important severe syndromes—pneumonia, sepsis, and meningitis—this review aimed to assess variability and appropriateness in pediatric beta‐lactam dosing recommendations for selected WHO AWaRe antibiotics based on the assumption that these were severe infections in hospitalized neonates and children. We collated dosing guidance from national and international pediatric formularies, searched for the most appropriate model for suggested drugs, and simulated target attainment in a real‐world setting by sampling underlying syndrome‐specific populations from point prevalence surveys.

## Methods

### Choice of beta‐lactam antibiotics and pharmacokinetic model selection

For pneumonia, sepsis, and meningitis, common beta‐lactam antibiotic/organism (drug/bug) combinations were extracted from the most recent English Surveillance Program For Antimicrobial Utilization And Resistance (ESPAUR) Report in 2018–2019.^13^ For pneumonia, the antibiotics selected were amoxicillin‐clavulanate (co‐amoxiclav), ampicillin/sulbactam, and benzylpenicillin (penicillin G). Oral co‐amoxiclav was also evaluated for step‐down treatment. For sepsis the following antibiotics were chosen: cefotaxime, ceftazidime, ceftriaxone, co‐amoxiclav, piperacillin‐tazobactam, and meropenem. For meningitis, cefotaxime, ceftriaxone, and meropenem were selected.

For each drug a pediatric pharmacokinetic (PK) model was identified from our recently published systematic review.^14^ This review suggested an evidence grading based on the data and modeling presented. Briefly, a scoring system was developed to rate the conducted PK assessment through the pharmacokinetic‐pharmacodynamic (PKPD) dosing evidence score. On top of the score, extracted publications were then assessed regarding the underlying studies’ quality to derive the overall quality‐of‐evidence rating, which was summarized as strong, intermediate, or weak strength of recommendation. The literature search was limited to articles published on pediatric PK studies for AWaRe antibiotics, and the search was conducted in PubMed. A graphical overview of the scoring system can be found in **Figure** **S1**. The search was repeated for the subsequent time frame May 2018 to October 2020 to include more recently published literature.

For the current assessment, PK models were chosen based on their quality‐of‐evidence rating. Where pediatric models were not available, adult models were scaled down to pediatric populations by including allometric scaling and maturation functions.^15^, ^16^, ^17^, ^18^ If multiple models of the same evidence score were found eligible, a decision was made taking size of the modeled cohort, published goodness‐of‐fit criteria, and the included significant covariates available in the point prevalence data set into account. For the models designated for our simulations, model code was retrieved from the publication’s supplementary information. If model code was not published, the authors were contacted to share the original code.

### Neonatal and pediatric demographic population development

Demographics for a population of hypothetical neonates, infants, and children with pneumonia, sepsis, and meningitis were then generated. Real demographics were sampled using R (version 4.0.2; R foundation for statistical computing, Vienna, Austria) from three different data sets: a one‐day point prevalence survey (PPS) on antibiotic prescription, which was collected from the Global Antimicrobial Resistance, Prescribing and Efficacy in Neonates and Children (GARPEC) Network;^8^ the Global Point Prevalence Survey on Antimicrobial Consumption and Resistance (Global PPS) network;^8^ and the Antibiotic Resistance and Prescribing in European Children (ARPEC) project.^19^ Patients aged < 19 years receiving at least one antibiotic on the day of the respective survey were included.

In total, 116 hospitals from 26 countries participated in the GARPEC PPS between 2015 and 2017. The Global PPS survey, conducted between October 2014 and November 2015, included 335 hospitals from 53 countries. ARPEC was carried out in 18 centers across 11 countries. Patients were assigned to pneumonia, sepsis, or meningitis based on the documented reason for starting antibiotics in the respective survey databases. From each data set the demographic covariates age, weight, and sex were extracted; for neonates postnatal age and gestational age were extracted and postmenstrual age calculated.

The individual patient’s age‐dependent typical creatinine concentration was derived from the equation by Ceriotti *et al*.^20^ This method has previously been used to model age‐adjusted creatinine in PK studies.^21^, ^22^, ^23^ Typical albumin concentration was calculated based on the patient's postmenstrual age in line with previous publications.^24^, ^25^ The demographics derived from the PPS data sets can be seen as real‐world cases, and the formed subpopulations represent the actual target population for each treatment indication.

### Systematic search for dosing guidelines

Pediatric formularies were searched to identify the dosing regimens for each antibiotic to treat each clinical infection. The following national and international formularies were chosen, in order to cover the range of regimen per drug, that are used globally: British National Formulary for Children,^26^ Dutch/German Database for Pediatric Dosing (Kinderformularium),^27^ German Pediatric Infectious Diseases Society Handbook,^28^ Swiss Database for Dosing Medicinal Products in Pediatrics (SwissPedDose),^29^ Indian National Center for Disease Control guidance,^30^ Nelson’s Pediatric Antimicrobial Therapy (Bradley *et al*.),^31^ Manual of Childhood Infections (Blue Book),^32^ Report of the Committee on Infectious Diseases (Red Book),^33^ and the WHO Pocket Book.^34^

For the three infectious diseases, pneumonia, sepsis, and meningitis, dosing guidance was extracted from each formulary. Additionally, the minimum and maximum regimen were listed alongside the WHO expert consensus regimen^5^ aiming to display a median regimen across all formularies. Dosing information was collected for the following pediatric age groups: neonates (≤ 28 days); infants/children (>28 days–12 years) and adolescents (>12 years).

### Pharmacokinetic‐pharmacodynamic analysis

Simulations for the chosen drugs’ PKPD relationships were conducted from the chosen models using NONMEM 7.4 (version 7.4; ICON Development Solutions, Ellicott City, MD). Post‐simulation processing and graphical evaluations were performed in R. For pneumonia the European Committee on Antimicrobial Susceptibility Testing (EUCAST) nonspecies‐related drug‐specific sensitivity and resistance breakpoints were compared with target attainment, whereas for sepsis and meningitis the breakpoints for Enterobacterales were used. Histograms of some the following example organisms drawn from the EUCAST MIC distribution database were displayed for comparison: *Streptococcus pneumoniae* and *Staphylococcus aureus* for pneumonia and *Escherichia coli, Klebsiella pneumoniae*, and *Pseudomonas aeruginosa* for sepsis and meningitis. The chosen organism/antibiotic (bug/drug) combinations and extracted common, minimum, and maximum dosing recommendations were used to generate probability of target attainment (PTA) simulations within the targeted subpopulation by using the PKPD model with the highest level of evidence in this setting. The evaluated targets were dependent on the respective simulated drugs. Susceptibilities were displayed as MIC distributions; breakpoints reflecting sensitive and resistant isolates were derived from the EUCAST database for the respective microorganisms. For simulations in each clinical condition 10,000 demographic sets were sampled with replacement from the GARPEC / Global PPS / ARPEC subpopulation data sets. For clinical markers creatinine, creatinine clearance, and albumin that were derived from age‐dependent functions, a variance of 10% around the calculated typical values was introduced.

### Target attainment analysis

Pharmacodynamic (PD) analysis was carried out through PTA assessment from the simulations. Targets were chosen depending on the pharmacodynamics of the investigated drug / drug class. As suggested by Mouton *et al*.^35^ the fraction of time above MIC is seen as the appropriate target for beta‐lactam antibiotics. Free, unbound concentrations were used to evaluate the PKPD target. For ceftriaxone, free concentration accounting for nonlinear concentration‐dependent protein binding was assumed.^36^ For the other agents the following fraction unbound was assumed: amoxicillin 83%,^37^ ampicillin 80%,^38^ benzylpenicillin 45%,^39^ cefotaxime 70%,^39^ ceftazidime 83%,^40^ meropenem 98%,^37^ and piperacillin 70%.^37^ In line with current guidance for severe hospitalized patients, an overall beta‐lactam PKPD target of 100% fT > MIC within a 24‐hour time frame at steady state was chosen.^41^ Further investigation was performed looking at 50% fT > MIC for less severe infections that can be found in the community setting and 100% fT > 4× MIC when a critically ill population is targeted.^42^

Results from the target attainment analysis were compared graphically and numerically with isolate‐specific MIC distributions from EUCAST clinical breakpoint reports. For sepsis and meningitis sensitive and resistant breakpoints reflecting Enterobacterales were added. In pneumonia nonspecies‐related breakpoints were chosen.

### Toxicity analysis

For the simulated drug toxicity, cutoffs were extracted from the literature. A general neurotoxicity threshold of 316 mg/L was used for beta‐lactams in line with findings by Imani *et al*.^12^ For cephalosporins a threshold of 35 mg/L, as reported by Huwyler *et al*.^43^ for cefepime, was chosen. Cefepime is seen as the cephalosporin with the highest risk for causing neurotoxicity. Other drug‐specific targets reported in literature for trough concentrations correlated with a proconvulsive risk that were tested are 64mg/L for meropenem and 157 mg/L for piperacillin when combined with tazobactam.^12^

## Results

### Demographic data sets

After cleaning and combining the three point‐prevalence data sets GARPEC, Global PPS and ARPEC, subpopulations that were treated for pneumonia, sepsis, and meningitis consisted of 2,932, 2,269, and 1,358 individuals, respectively, with age ranging from 23 weeks postmenstrual age to 18 years postnatal age (PNA), and weight ranging from 0.31 kg to 95.5 kg.

**Figure 1:**
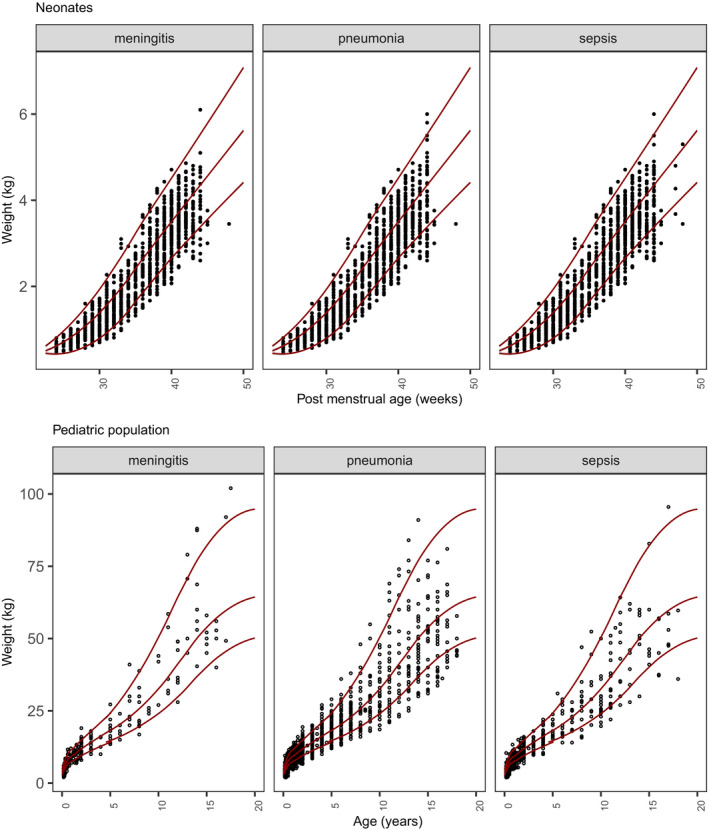
Weight compared with age for the three simulated subpopulations: pneumonia, sepsis, and meningitis. Black dots are single subjects filtered from GARPEC / ARPEC / Global PPS. Gray lines represent the median, 3rd, and 97th percentiles from demographic surveys by Fenton *et al*., CDC (Centers for Disease Control and Prevention), and WHO (World Health Organization).

**Figure ****1** shows the age‐related weight distribution for each subpopulation with an additional panel showing the included neonates. Each graph also shows the typical weight for age distribution published for preterm and term neonates by Fenton *et al*.,^44^, ^45^ for up to 5‐year‐olds by the WHO^46^ and for 5‐year‐olds to 18‐year‐olds by the Centers for Disease Control and Prevention (CDC).^47^

### Dosing regimen

**Table ****S1** summarizes the dosing recommendations for the different age groups (from preterm neonates to adolescents) extracted from summaries of product charcteristics (SmPCs) and formularies, together with the common, minimum, and maximum regimen.

Within the three different infections, pneumonia, sepsis, and meningitis, 6/120 (5%), 10/180 (6%), and 40/90 (44%) of the indivdual dose recommendations, respectively, were specific recommendations for the evaluated disease. Overall, for 88/390 (23%), dosing recommendations were lacking for the respective age group, drug, and formulary.

### Selected PK models for simulation

The systematic literature search and evidence grading resulted in five models with a mean dose evidence score (DES) of 8 (7–10). The overall quality of evidence (QoE) was rated strong (n = 2) or intermediate (n = 3). The beta‐lactams benzylpenicillin, intravenous co‐amoxiclav, piperacillin‐tazobactam, cefotaxime, and meropenem were well described by Lonsdale *et al*.^15^ (DES = 7, strong QoE) across the entire age range. Oral co‐amoxiclav was simulated according to the model by deVelde *et al*.^48^ (DES = 10, intermediate QoE); ampicillin‐sulbactam was modeled according to Soto *et al*.^49^ (DES = 9, intermediate QoE); ceftriaxone according to Standing *et al*.^36^ (DES = 10, intermediate QoE); and ceftazidime by using the model from Li *et al*.^50^ (DES = 7, strong QoE). Selected models are summarized in **Table** **S3**.

### Evaluating probability of target attainment

**Figure 2:**
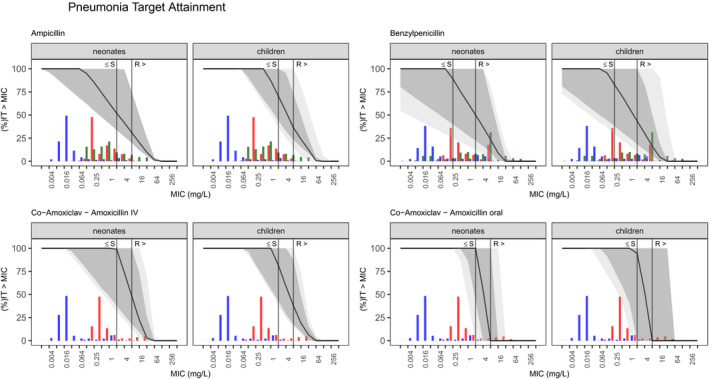
Probability of target attainment (100%fT > MIC) for simulated dosing regimen in the pneumonia subpopulation. Solid line, median for common regimen; dark gray area, 90% prediction interval for common regimen; light gray area, 5th percentile of minimal regimen to 95th percentile of maximal regimen. Colored histograms refer to the MIC distribution for common pathogens *Haemophilus* *influenzae* (red), *Streptococcus Pneumoniae* (blue) and *Staphylococcus aureus* (green) according to EUCAST (European Committee on Antimicrobial Susceptibility Testing). The gray solid vertical line represents the nonspecies‐specific breakpoint values for each drug to guide empiric treatment. fT > MIC, fraction of time of the free drug concentration being above the MIC; IV, intravenous; MIC, minimum inhibitory concentration; R, resistant breakpoint; S, sensitive breakpoint.

**Figure 3:**
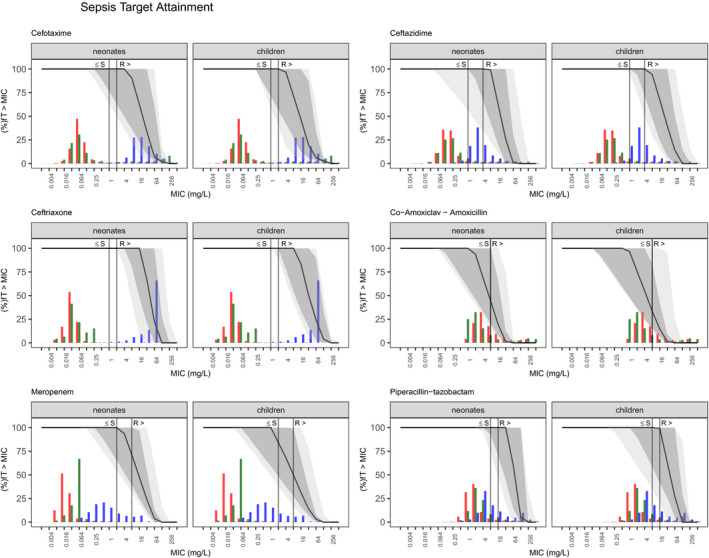
Probability of target attainment (100%fT > MIC) for simulated dosing regimen in the sepsis subpopulation. Solid line, median for common regimen; dark gray area, 90% prediction interval for common regimen; light gray area, 5th percentile of minimal regimen to 95th percentile of maximal regimen. Colored histograms refer to the MIC distribution for common pathogens *Escherichia* *coli* (red), *Klebsiella pneumoniae* (green), and *Pseudomonas aeruginosa* (blue) according to EUCAST (European Committee on Antimicrobial Susceptibility Testing). The gray solid vertical line represents Enterobacterales breakpoints for each drug to guide empiric treatment. fT > MIC, fraction of time of the free drug concentration being above the MIC; MIC, minimum inhibitory concentration; R, resistant breakpoint; S, sensitive breakpoint.

**Figure 4:**
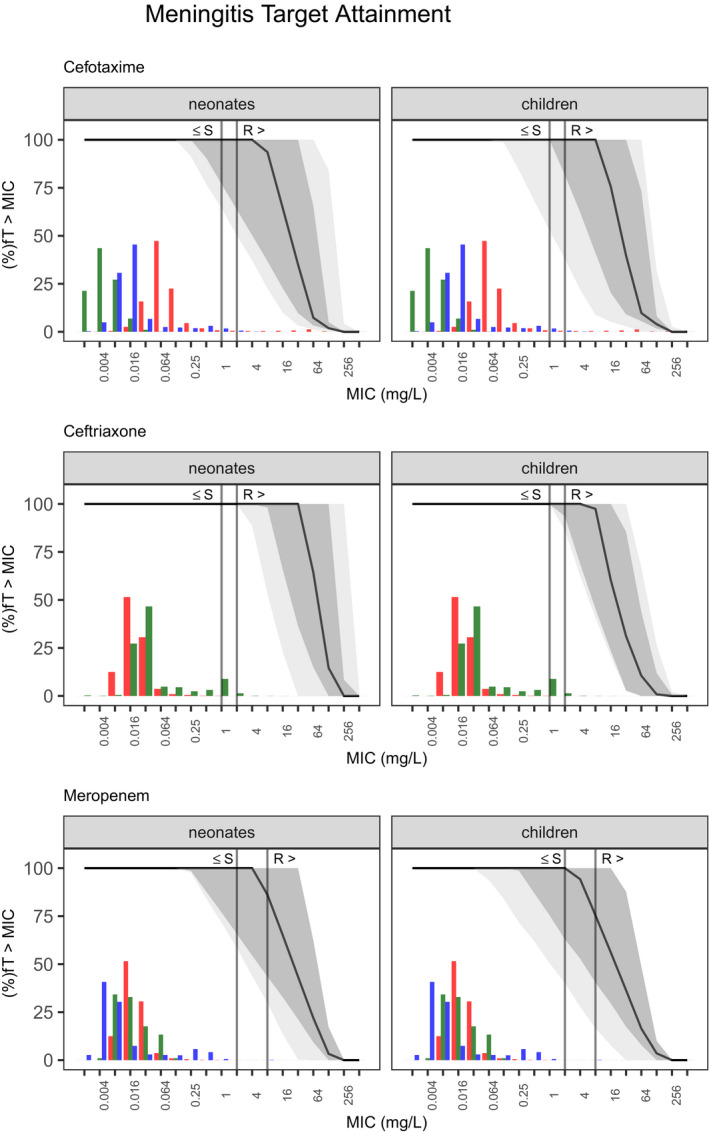
Probability of target attainment (100%fT > MIC) for simulated dosing regimen in the meningitis subpopulation. Solid line, median for common regimen; dark gray area, 90% confidence interval for common regimen; light gray area, 5th percentile of minimal regimen to 95th percentile of maximal regimen. Colored histograms refer to the MIC distribution for common pathogens *Neisseria* *meningitidis* (green), *Streptococcus pneumoniae* (blue) and *Escherichia* *coli* (red) according to EUCAST (European Committee on Antimicrobial Susceptibility Testing). The dark gray solid vertical line represents Enterobacterales breakpoints for each drug to guide empiric treatment. fT > MIC, fraction of time of the free drug concentration being above the MIC; MIC, minimum inhibitory concentration; R, resistant breakpoint; S, sensitive breakpoint.

Common, minimum, and maximum dosing was simulated for each drug using the chosen highest evidence models. **Figure** **2** shows the target attainment results for pneumonia, **Figure** **3** for sepsis, and **Figure** **4** for meningitis. Dark shaded areas show the 90% prediction interval across the simulated subpopulation for the common regimen, with the solid black line showing the population mean. Light shaded areas show the prediction interval stretching from the 5^th^ percentile of the minimum dose to the 95^th^ percentile of the maximum dose. The simulation results were explored graphically for each disease against the chosen isolates EUCAST MIC distribution through the presentation of colored histograms representing the respective isolates distribution. The EUCAST sensitive and resistant breakpoints for Enterobacterales are shown as solid gray lines within the sepsis and meningitis assessments. For pneumonia the EUCAST nonspecific sensitive and resistant breakpoints are shown.

The predicted MIC at which the targets 100% fT > MIC and 100% fT > 4× MIC are crossed is reported for each antibiotic alongside the EUCAST epidemiological cutoff value and empiric target MIC values for the considered isolates (**Table** **S2**).

**Figure 5:**
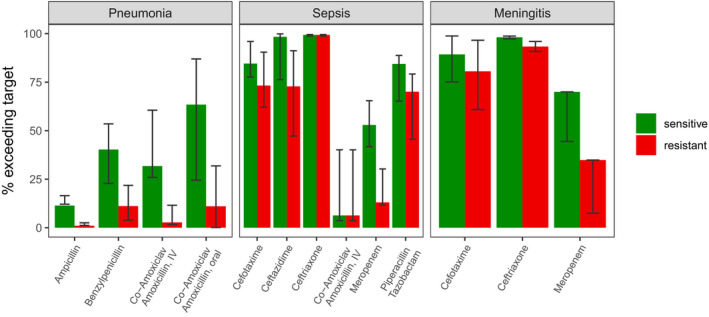
Coverage calculated as percentage of individuals above the PKPD target of 100%fT > MIC for each simulated drug for the three different syndromes. Each bar represents the coverage of the common regimen, with the error bar showing results of the minimal to maximal simulated regimen. Bars are split up by sensitive and resistant MIC breakpoints for Enterobacteraes (sepsis and meningitis) or nonspecific (pneumonia). fT > MIC, fraction of time of the free drug concentration being above the MIC; IV, intravenous; MIC, minimum inhibitory concentration; PKPD, pharmacokinetic‐pharmacodynamic.

The percentage of patients reaching the 100% fT > MIC PKPD target, when sensitive and resistant breakpoints for Enterobacterales (sepsis and meningitis) or nonspecies related (pneumonia) are considered is summarized in **Figure** **5**.

### Toxicity

**Figure 6:**
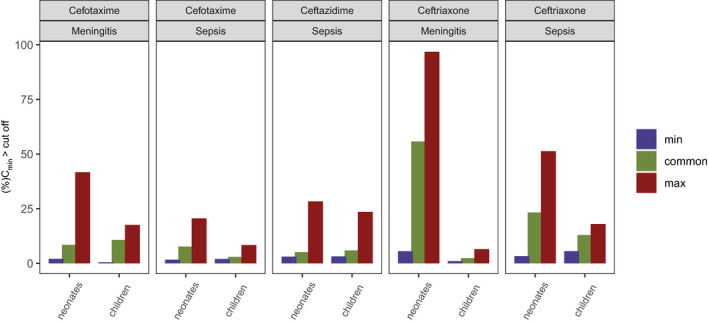
Percentage of subjects above toxicity threshold (>35 mg/L) for cephalosporins and the respective syndrome. Plots are grouped by age. Each bar represents the fraction of simulated individual with a trough concentration at steady state above the toxicity threshold. min, minimal; max, maximal.

The probability of trough concentrations crossing the literature‐reported toxicity thresholds is depicted in **Figure** **6**. The trough concentration at steady state was evaluated for every individual, and the proportion of individuals crossing the threshold is displayed. Only the risk of toxicity for the different cephalosporin regimens to cross the reported cefepime trough threshold of 35 mg/L is shown, as other drug classes did not notably exceed the toxicity threshold for any of the simulated regimens.

## Discussion

In our analysis we focused on commonly used beta‐lactam antibiotics featuring in the WHO EML for children.^5^ Point prevalence surveys have revealed heterogeneity in dosing across and within countries,^51^ and we found this to be reflected in varying dosing recommendations provided by national and international formularies.

Due to developmental differences in drug handling (mainly lower clearance),^52^ neonates are usually given lower per‐killogram doses; hence we split assessment of target attainment between neonates and children. Figures 2, 3, 4 show each drug’s PKPD target attainment trajectory across MIC values for 100%fT > MIC in neonates and children. The simulated common dosing regimen showed third generation cephalosporin dosing guidelines are generally appropriate (**Figure** **5**). Sufficient coverage was achieved for ceftriaxone when used in empiric sepsis and meningitis treatment, with at least 90% of the simulated patient population being covered for sensitive *Enterobacterales*. Cefotaxime also showed good coverage in both severe syndromes, yielding at least 80% of patients reaching the PKPD target considering sensitive *Enterobacterales* MICs (**Figure** **5**). When MIC targets reflecting resistant breakpoints are chosen, ceftriaxone still performs well.

Considering the resistant breakpoints for *Enterobacterales*, ceftazidime reached at least 70% coverage in sepsis treatment, and cefotaxime covered at least 70% of the population for sepsis and meningitis, whereas meropenem failed to successfully reach adequate concentrations in both diseases. This is largely due to the high empiric MIC cutoff of 2mg/L reflecting the fact that target attainment in settings with borderline carbapenem resistance would likely require higher meningitis doses even when treating sepsis and the use of an overall beta‐lactam PKPD target of 100%fT > MIC. Carbapenems show a higher postantibiotic effect compared with other beta‐lactams. A target of 20%fT > MIC has been shown to be bacteriostatic in *in vitro* models, with 40%fT > MIC resulting in bactericidal effects.

While piperacillin‐tazobactam also achieved sufficient levels in more than 75% of the pediatric population regarding sensitive breakpoints (**Figure** **5**), isolate‐specific epidemiological cutoff values, representing the critical epidemiological MIC that covers all MICs of wild‐type isolates values, are, however, higher than this nonspecies‐specific value. Coverage for the resistant *Enterobacterales* breakpoint of 16 mg/L only accounts for 70% (79−46%). A re‐analysis of the microbiology data from the MERINO trial showed that infection with extended‐spectrum beta‐lactamase organisms, while “sensitive” (with MICs below 16 mg/L), were still associated with increased mortality,^53^ with insufficient target attainment a possible cause.

In pneumonia, none of the studied scenarios led to adequate coverage when considering target attainment with sensitive nonspecies‐related MIC values (**Figure** **5**). However, taking commonly detected species into account, pneumonia is dominated by gram‐positive strains like *S. aureus* and *S. pneumoniae*, which generally show low MICs. When examining the EUCAST MIC distributions in **Figure** **2** describing these two strains plus the gram‐negative non–type *b* *Haemophilus* *influenza,* ^54^ co‐amoxiclav sufficiently covers the gram‐positive strains, whereas penicillin G and ampicillin‐sulbactam cover the median of the simulated population at best. When looking at sepsis, EUCAST MIC distributions for *E. coli* and *K. pneumoniae* are fairly well covered by cephalosporins and meropenem. *Pseudomonas*, on the other hand, was less sensitive to most of the studied drugs (**Figure** **3**). *Pseudomonas* generally causes sepsis in hospitalized immunocompromised children and hence in this setting drug choices and combination therapies with better *Pseudomonas* coverage would be more appropriate than increasing the doses of the agents discussed here.

Our findings are in line with Hartman *et al*.,^55^ who recently reviewed the pharmacokinetics and target attainment of antibiotics in critically ill children and reported that target attainment in this patient group is suboptimal. They found that for highly monitored substances like glycopeptides and aminoglycosides, there are a large number of publications available. In fact, 41 vancomycin and 53 gentamicin studies have also been detected in our previous grading evidence review with a median dose evidence score of 4 (2–11) and 3 (1–10), respectively. For most beta‐lactams, however, the available information is sparse or completely lacking for children, especially when narrowing it down to the critical care setting. Throughout the literature, the PKPD adequacy for beta‐lactams in pediatric patient populations has most commonly been assessed using the following targets: penicillins 40–50%fT > MIC, cephalosporins 50–70%fT > MIC, and carbapenems, which display postantibiotic effects 40%fT > MIC.^56^, ^57^, ^58^

A current analysis by van Donge *et al*.^59^ evaluated common amoxicillin dosing in neonates by simulating these more conservative PKPD targets along with higher 100% fT > MIC or 100% fT > 4× MIC and the probability of reaching neurotoxic exposures. Here, low PKPD targets were well covered with common regimens. Higher targets, when a PTA > 90% was aspired, were failed by all regimens, with the highest simulated regimen expecting exposure above the peak plasma concentration (C_max_) toxicity threshold of 140 mg/L.

We focused on EUCAST MIC reporting, but empiric treatment decisions are dependent on local resistance patterns and may deviate from what is simulated with the nonspecies‐related MICs, globally. Figures 2, 3, 4 are depicted with common MIC ranges and can be used for local dosing regimen design based on local MIC distributions. The selection of a PKPD target in antibiotic treatment depends on the severity of the targeted disease, focus of the infection, individual patient factors, and local resistance patterns.^60^ As beta‐lactams show time‐dependent bactericidal effects, the fraction of time that the free concentration is above a multiple of the MIC should be targeted. For our simulations we focused on an overall beta‐lactam PKPD target of 100%fT > MIC.

Bactericidal effect for beta‐lactam classes is detectable for %fT > MIC as low as 40% in carbapenems, and 50–70% in cephalosporins and penicillins.^41^ In critical care the 100%fT > MIC target, however, resulted in favorable outcomes according to the defining antibiotic levels in intensive care unit patients (DALI) trial.^42^ The desired PD targets, however, still remain to be elucidated across different routes of administration and patient populations.^61^ The use of continuous infusion regimen, as studied in the Beta‐Lactam Infusion Group Study (BLING) I‐III for adult critical care patients, is often not feasible in neonatal and pediatric settings, where intravenous accessibility, volumes, and compatibilities have to be taken into account when performing infusion management.^62^

Thus, across the heterogenous severity stages, which are seen in the simulated syndromes, a PKPD target of 100% fT > MIC seems sensible for achieving overall clinical efficacy, with the option to accept lower coverage between 50 and 100% fT > MIC (Figures S2–S4) in less severe infections and increasing the target to 100% fT > 4× MIC (Figures S5–S7) in critically ill patients cared for in pediatric intensive care unit and neonatal intensive care unit settings. These PKPD targets are used with simulations of plasma concentrations as surrogates for the less accessible focuses in meningitis and pneumonia, cerebrospinal fluid, and epithelial lining fluid. A drug’s ability to cross into these deeper compartments correlates with its physicochemical properties enabling the passage through membranes, transporter affinities, and physiological conditions that are also influenced by the pathology of infectious disease, such as pH changes and membrane permeabilities.^63^ More insight is needed to understand how maturation influences a drug's distribution to the site of infection and the ability of plasma concentrations to serve as surrogates for this.

Similarly, our simulation population includes creatinine concentrations used as a covariate in some of the selected models, varying around the typical age‐related value. Our simulations therefore represent target attainment overall.

To depict the influence acute kidney injury (AKI) and augmented clearance would have on the performed simulations, we assessed models including the creatinine covariate. Simulated populations with at least a 50% increase from baseline creatinine, in line with the Kidney Disease: Improving Global Outcomes (KIDGO) criteria diagnosing AKI,^64^ and a 50% decrease in creatinine for augmented renal function are given in **Figure** **S8**. This highlights the corresponding improvement or worsening in target attainment with AKI or augmented renal function, respectively.

The formularies used in this review were both national (four European, two US, one Indian) and international. The summaries of product charcteristic (SmPC) recommendation for each drug was also studied. Despite consulting national experts, it was not possible to find pediatric formularies from Brazil, China, the Russian Federation, or South Africa that were clearly endorsed nationally, which is in line with a previous analysis by Mathur *et al*.^9^ We therefore acknowledge that the presented dose collection is strongly influenced by European and North American dosing guidance. The consulted formularies each state dosing recommendations for almost every drug that was selected. However, there are gaps regarding age‐specific and disease‐specific recommendations. The lack of information is most marked for neonates, whereas adult information is often used for adolescents and thus is available.

A trend in diversity of dosing recommendations, age‐banding, and weight‐based dosing can be found in older drugs like penicillin G. For this particular drug, doses are historically stated in "IU" (international units) for some countries and "mg" (milligrams) in others. For co‐amoxiclav, some formularies state volume of a specified strength of suspension rather than a drug‐specific dose amount. This was also found for the other fixed‐combination beta‐lactam and beta‐lactamase inhibitor preparations, where it is not always clearly stated whether the dose amounts in the formularies refer to the beta‐lactam component or the combination.

Through our previous study to grade evidence in pediatric antibiotic PK reporting,^14^, ^65^ supplemented by an updated literature search, we identified the models used in the target attainment simulations. All models used in the simulations were rated with a dose evidence score between 7 and 10, with 12 being the maximal achievable score. The overall quality of evidence was rated strong or intermediate, with intermediate rating rather than strong due to most studies being conducted in a single‐center setting, without additional data for validation. A key criterion for choosing appropriate models was that covariate parameterization allowed for extrapolation across the neonatal and pediatric age range, which is often not possible for models developed in subpopulations with empirical covariate structures.^66^

Models that are scalable across the entire pediatric age range are still lacking for many antibiotics. For beta‐lactams a recent study by Lonsdale *et al*.^15^ developed a maturation function that is able to describe the maturation of the mostly renal clearance mechanisms in beta‐lactams from neonates to elderly patients. For other drug classes similar investigations are still missing. In adults, PK studies on antibiotics have recently focused increasingly on determining elimination and distribution mechanisms in the disease and syndrome specific context. Many adult PK studies are investigating specific patient subpopulations with, for example, pneumonia or intra‐abdominal infections, and exploring the effects of supportive therapies, such as dialysis or extra corporeal membrane oxygenation, on PK.

A limitation of our work is that we did not consider subpopulations of the pediatric group. In pediatrics, special subpopulation PK is still not well characterized, as is the PK for most fragile pediatric subpopulation of preterms, neonates, and the influence of prematurity for low and very‐low birthweight neonates.^67^, ^68^ The influence of nutritional status is also lacking for most PK studies conducted in pediatrics, but is important to inform dosing decisions when treating infections in underweight and malnourished children.^69^, ^70^, ^71^ Children treated in intensive care, and particularly those requiring renal replacement therapy and extra corporeal membrane oxygenation, may need altered dosing.^72^, ^73^

The evaluated toxicity simulations show that for the cephalosporins, the increased meningitis dosing may be associated with an increased risk of neurotoxicity (**Figure** **5**). Apart from cephalosporins, other drug classes did not show the risk of neurotoxicity in the studied scenarios and were therefore not reported in the graphic. Beta‐lactam neurotoxicity is not well studied and is difficult to distinguish from other neurological symptoms that can occur when treating infectious disease syndromes like bacterial meningitis. In the literature, mostly case reports are available. Imani *et al*.^12^ retrospectively evaluated beta‐lactam concentration–toxicity relationships through regression analysis, concluding that penicillins like piperacillin show neurotoxicity at trough concentrations as high as 361 mg/L. Animal studies report trough concentrations at 157 mg/L for piperacillin and 64 mg/L for meropenem toxicity.^74^ These targets were not or only barely (piperacillin) reached with the simulated carbapenems and penicillins. For cephalosporins, cefepime is seen as the most neurotoxic drug and therefore is studied more intensively compared with other drugs in this group. A threshold trough concentration of 35 mg/L is reported as threshold by Huwyler *et al*.^43^ and served as reference concentration for this analysis. It is anticipated that other cephalosporins bear lower risks of neurotoxicity. So far case reports dominate neurotoxicity reporting for cephalosporins other than cefepime, and thus the cefepime neurotoxic threshold serves as a surrogate for a regimen’s neurotoxic potential in this review. When treating severe infections involving organisms with high MICs, the potential risk of neurotoxicity needs to be balanced with treatment success and resistance suppression. For example, treating *Pseudomonas* infections using ceftazidime with a target of 100% fT > 4× MIC will result in an optimal trough exposure of 32 mg/L to cover resistant strain MICs of 8 mg/L. This highlights the need of antibiotic stewardship to consider personalized dosing based on patient and organism considerations.

Renal failure and drug accumulation due to impairment in the dominant elimination pathway is one of the main risk factors for beta‐lactam neurotoxicity. Findings for meropenem and piperacillin/tazobactam that relate free plasma trough concentration to the high *Pseudomonas* breakpoints suggest that minimal free drug concentration (fC_min_) above eight times the MIC will result in a less favorable risk–benefit ratio. This highlights the need to maintain a balance of sufficient exposure while avoiding unnecessarily high concentrations.

Overall, our review focused on beta‐lactam dosing on the WHO Essential Medicines List for Children (EMLc) Access and Watch antibiotic list. We aimed to find recommendations across the entire age range and thus did not choose antibiotics such as tetracycline or quinolones, which are not recommended in younger children and neonates due to their negative effects on joint, teeth, and ligament development. We also did not evaluate glycopeptides or aminoglycosides, as therapeutic drug monitoring is standard practice, and these classes are already thoroughly studied.

## Conclusion

The results of this review demonstrate that the high variability of dosing recommendations in national and international formularies impacts PKPD target attainment. While to some this may seem unsurprising, since beta‐lactams are generally thought to be drugs with a large therapeutic window, commonly used dosing schemes could routinely be set so high that variability does not in fact impact target attainment. However, a combination of increasing resistance and therefore MICs, and the often lack of regulatory licensing studies to set pediatric dosing, increasingly mean that choosing which guideline to follow is important, and should be informed by local sensitivity patterns and disease severity in the individual or population to be treated.

Pneumonia treatments show adequate target attainment in common gram‐positive infections. Here, co‐amoxiclav showed the best coverage across all extracted recommendations. Common regimens for cephalosporins in the treatment of sepsis and meningitis cover sensitive *Enterobacterales* infections reasonably well, and although more than 75% of the pediatric population is adequately covered in some instances, this leaves a small but significant proportion who are not. For intermediate‐to‐resistant MICs, *Enterobacterales* infections are covered in just over half of the population when using cephalosporins, with even lower target attainment for the penicillins and meropenem. Given that piperacillin‐tazobactam and meropenem are less likely to cause neurotoxicity than the cephalosporins, this suggests the need for reviewing current dosing recommendations in settings where MICs are typically in the intermediate range. The data presented here suggest that a “one‐size‐fits‐all” dosing recommendation may not be optimal in future and local dosing guidelines of AWaRe antibiotics may indeed be warranted to reflect variation in AMR patterns globally.

## Funding

S.G.’s postdoctoral fellowship is funded by Global Antibiotic Research and Development Partnership (GARDP). C.I.S.B. is funded by the National Institute for Health Research (NIHR) as an Academic Clinical Fellow. S.G., C.I.S.B., and J.F.S. have been supported by the National Institute for Health Research Biomedical Research Centre at Great Ormond Street Hospital for Children NHS Foundation Trust and University College London. C.I.S.B. is also supported by the NIHR Biomedical Research Centre based at Guy’s and St Thomas’ NHS Foundation Trust and King’s College London. The views expressed are those of the author(s) and not necessarily those of the NHS, the NIHR, or the Department of Health and Social Care. J.F.S. received a UK Medical Research Council fellowship (MR/M008665/1). M.C. is supported by core support from the Medical Research Council UK to the MRC Clinical Trials Unit (MC_UU_12023/22).

## Conflict of Interest

The authors declared no competing interests for this work.

## Disclaimer

As an Associate Editor of *Clinical Pharmacology & Therapeutics*, Joseph F. Standing was not involved in the review or decision process for this paper.

## Supporting information

Fig S1‐S8

Table S1

Table S2

Table S3
