# Supplementary material for: Variation in Target Attainment of Beta‐Lactam Antibiotic Dosing Between International Pediatric Formularies
Source: Clin Pharmacol Ther. 2021 Feb 28;109(4):958–70. doi: 10.1002/cpt.2180 (PMC8358626; doi:10.1002/cpt.2180)
Supplement: Supplementary file 1 — Fig S1‐S8 [file CPT-109-958-s002.pdf]

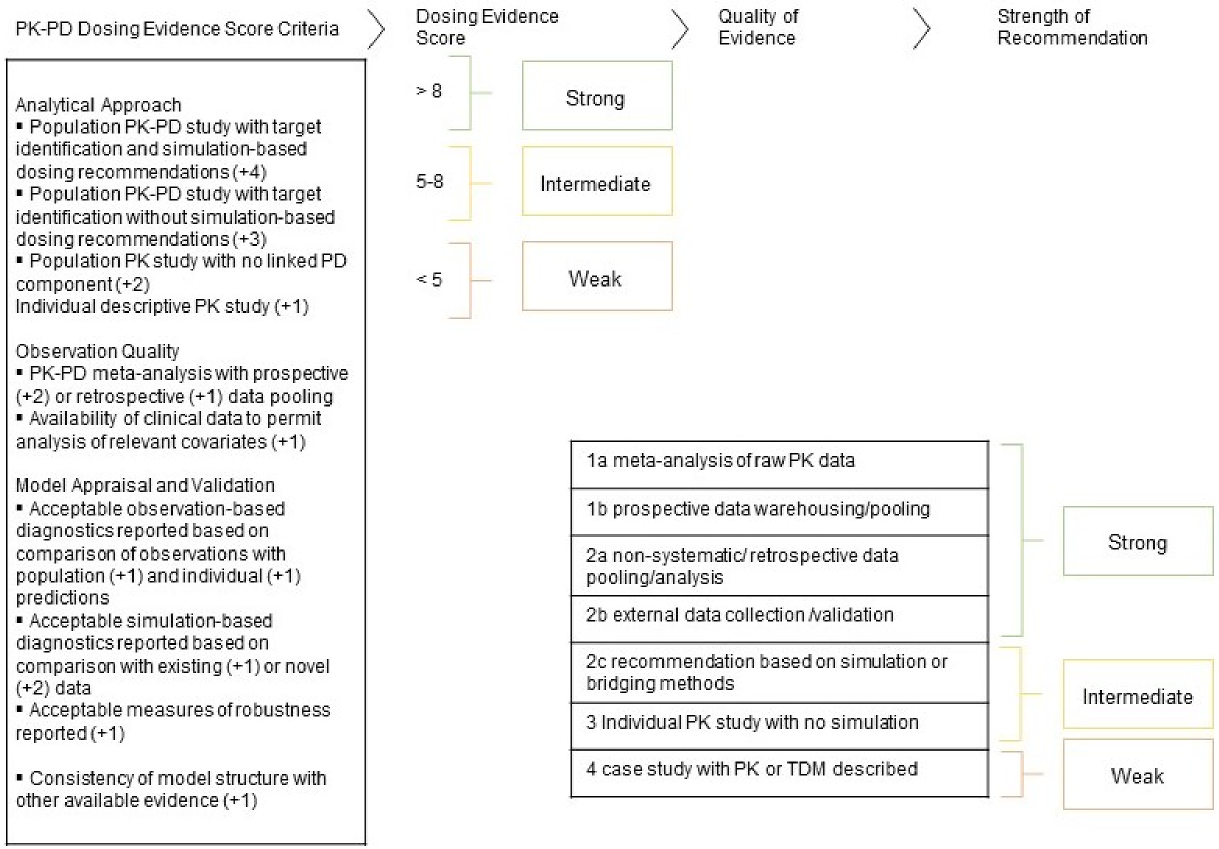

Figure S1: The GAPPS system uses a three part sequential assessment: PKPD Dosing Evidence Score (DES, numeric scoring 1 12), quality of evidence (QoE, levels of quality 1a 4) in summary the strength of recommendation in categories weak, intermediate, strong.

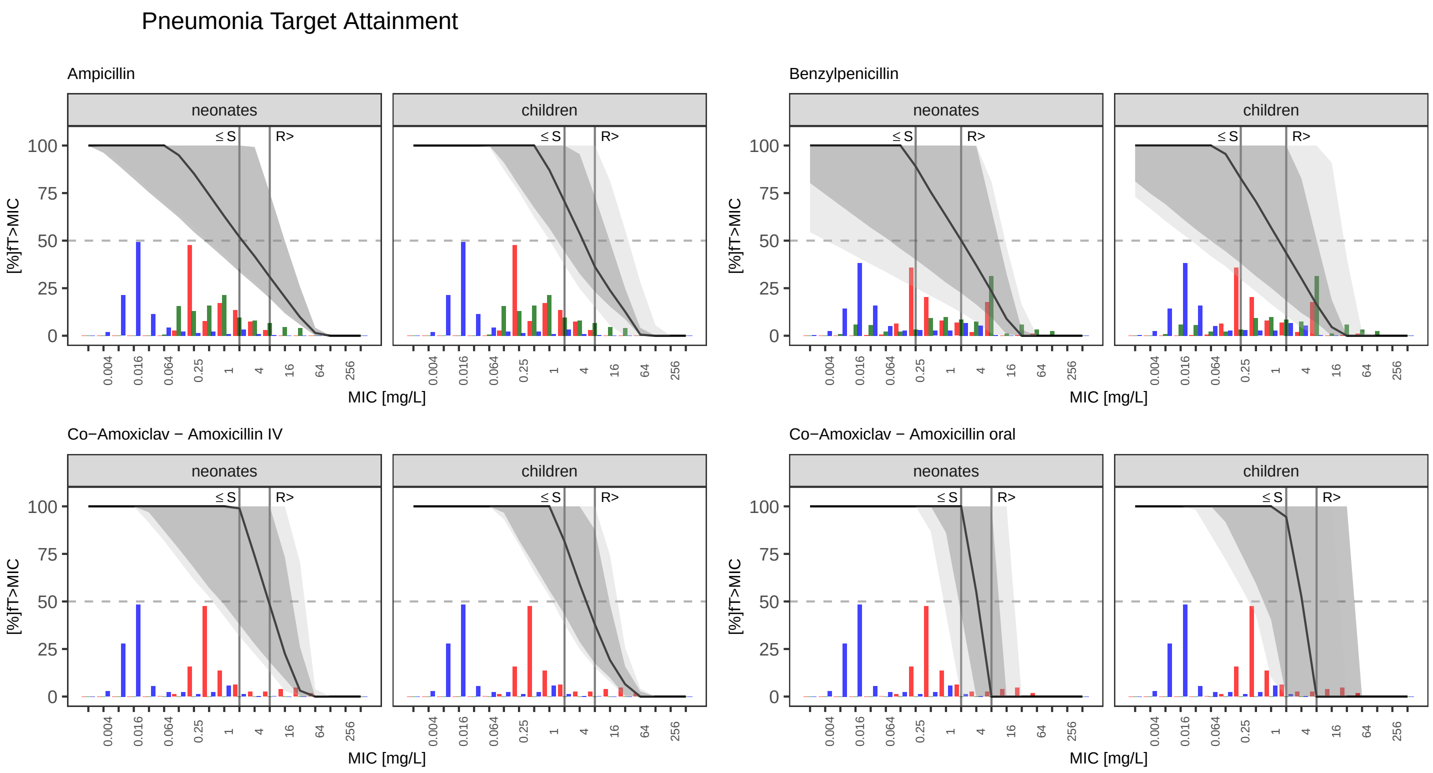

Figure S2: Probability of Target Attainment (50%fT>MIC) for simulated dose regimen in the pneumonia subpopulation. Solid line Median for common regimen; dark grey area 90% confidence interval for common regimen; light grey area 5th percentile of min regimen to 95th percentile of max regimen. Coloured histograms refer to the MIC distribution for common pathogens *Haemophilus influenzae* (red), *Streptococcus Pneumoniae* (blue) and *Staphylococcus aureus* (green) according to EUCAST. The grey solid vertical line represents the non-species-specific MIC values for each drug to guide empiric treatment. Dashed grey horizontal line shows 50% target.

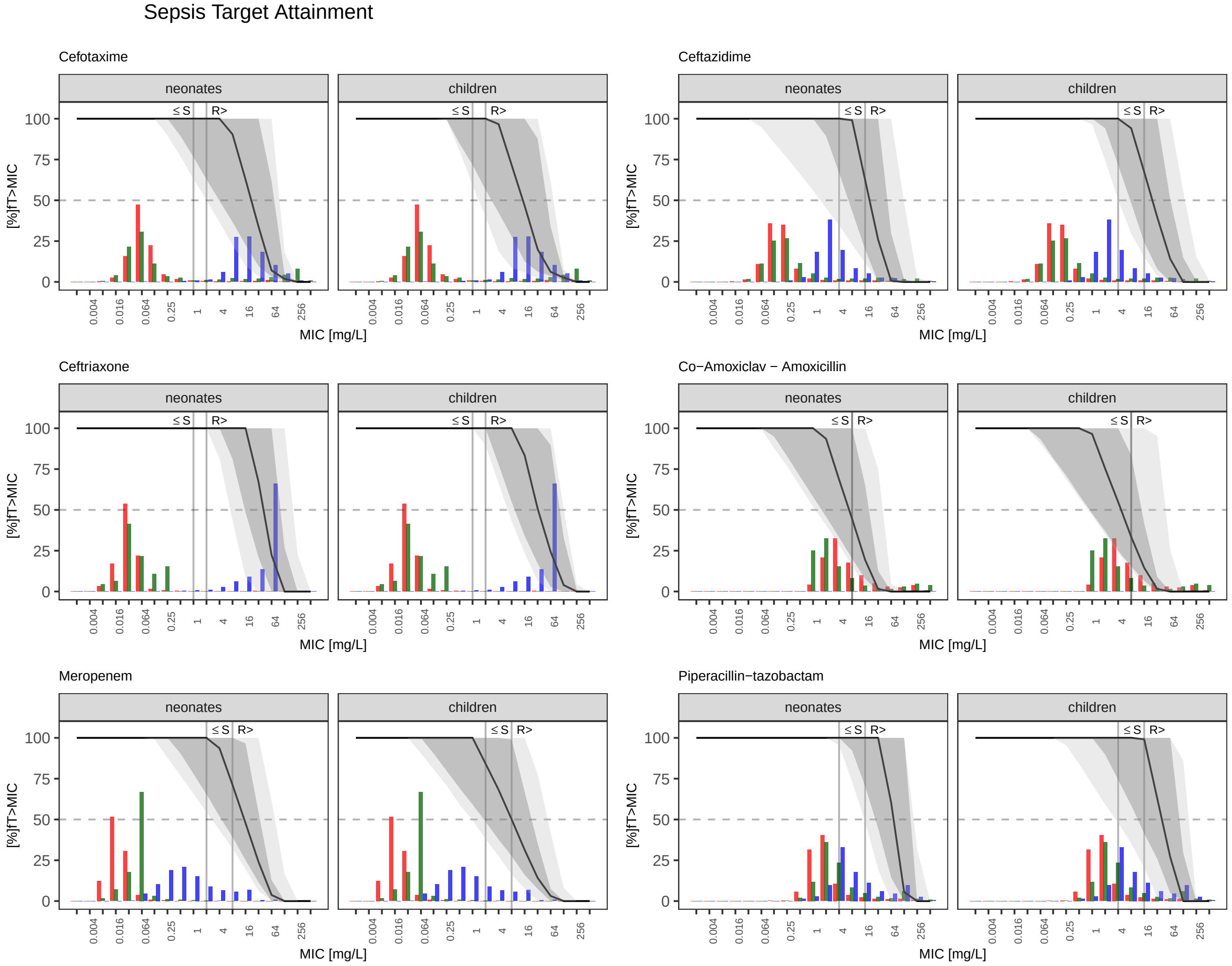

Figure S3: Probability of Target Attainment (50%fT>MIC) for simulated dose regimen in the sepsis subpopulation. Solid line Median for common regimen; dark grey area 90% confidence interval for common regimen; light grey area 5th percentile of min regimen to 95th percentile of max regimen. Coloured histograms refer to the MIC distribution for common pathogens were *E.coli* (red), *Klebsiella* species (green) and *Pseudomonas aeruginosa* (blue) according to EUCAST. The grey solid vertical line represents Enterobacterales breakpoints for each drug to guide empiric treatment. Dashed grey horizontal line shows 50% target.

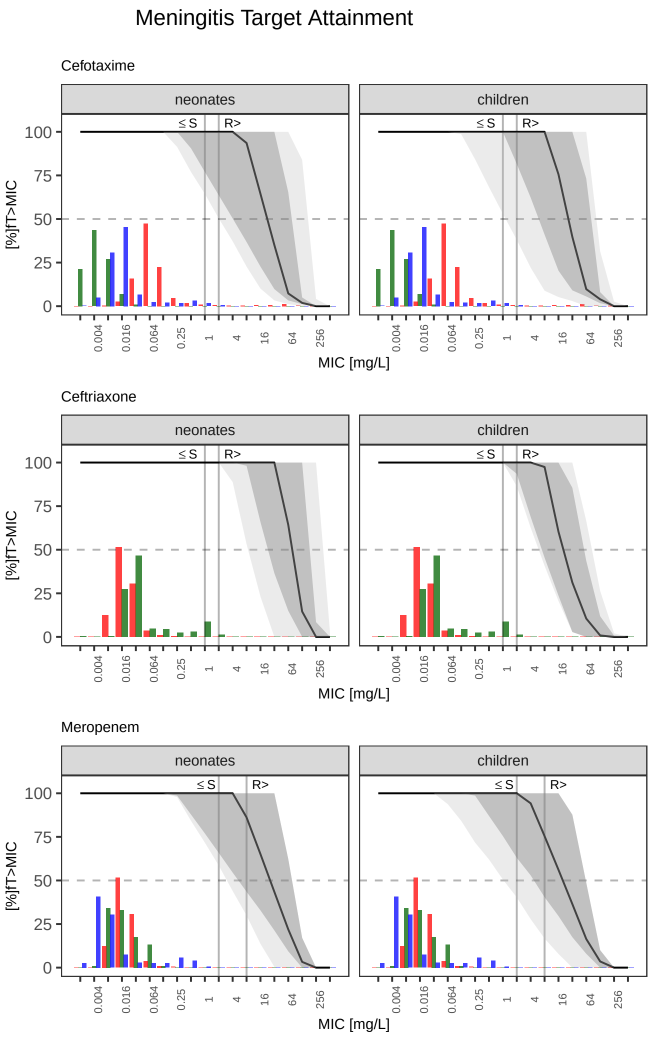

Figure S4: Probability of Target Attainment (50%fT>MIC) for simulated dose regimen in the meningitis subpopulation. Solid line Median for common regimen; dark grey area 90% confidence interval for common regimen; light grey area 5th percentile of min regimen to 95th percentile of max regimen. Colored histograms refer to the MIC distribution for common pathogens *Neisseria meningitidis* (green), *Streptococcus pneumoniae* (blue) and *E. coli* (red) according to EUCAST. The dark grey solid vertical line represents Enterobacterales breakpoints for each drug to guide empiric treatment. Dashed grey horizontal line shows 50% target.

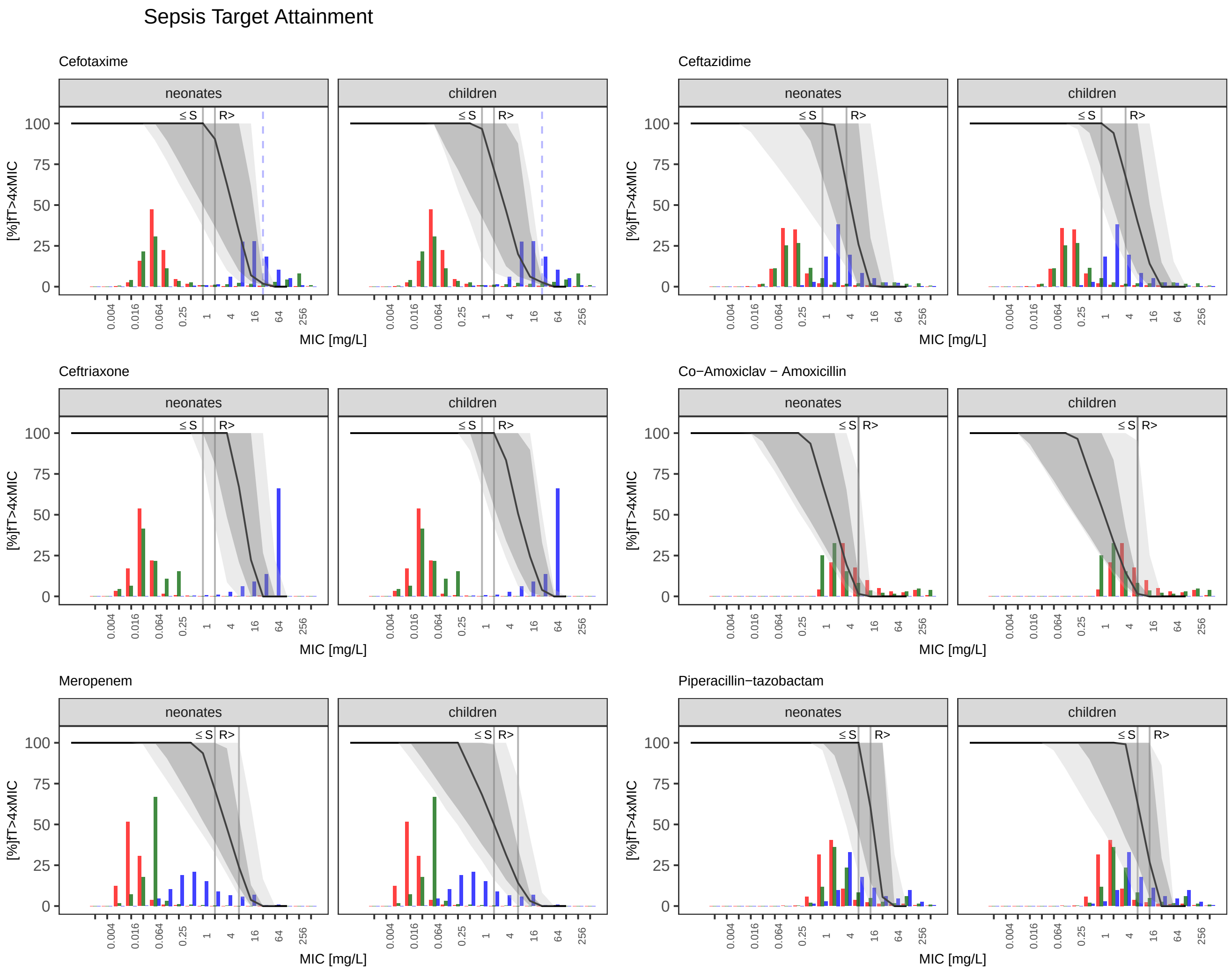

Figure S5: Probability of Target Attainment (100%fT>4xMIC)for simulated dose regimen in the pneumonia subpopulation. Solid line Median for common regimen; dark grey area 90% confidence interval for common regimen; light grey area 5th percentile of min regimen to 95th percentile of max regimen. Coloured histograms refer to the MIC distribution for common pathogens *Haemophilus influenzae* (red), *Streptococcus Pneumoniae* (blue) and *Staphylococcus aureus* (green) according to EUCAST. The dark grey solid vertical line represents the non-species-specific MIC values for each drug to guide empiric treatment.

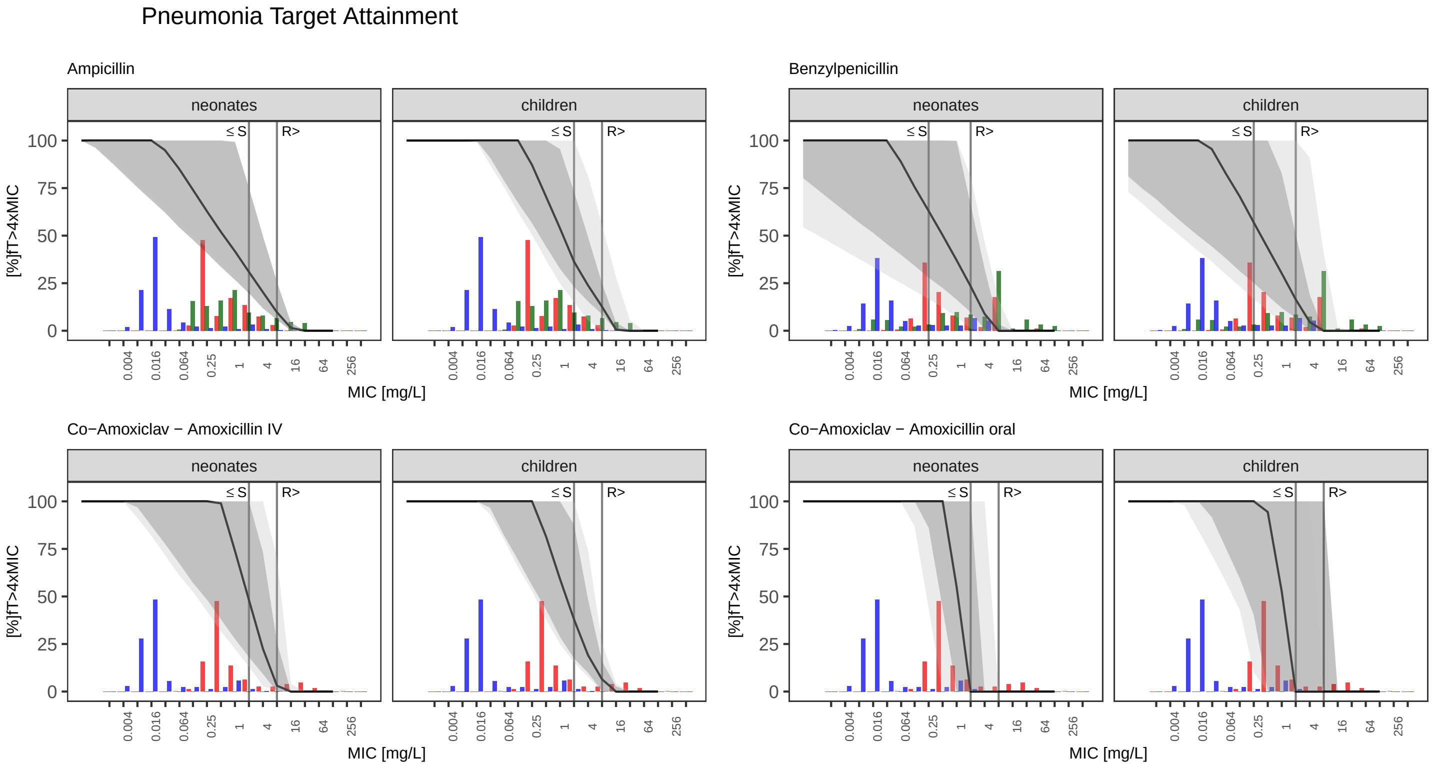

Figure S6: Probability of Target Attainment (100%fT>4xMIC) for simulated dose regimen in the sepsis subpopulation. Solid line Median for common regimen; dark grey area 90% confidence interval for common regimen; light grey area 5th percentile of min regimen to 95th percentile of max regimen. Coloured histograms refer to the MIC distribution for common pathogens were *E.coli* (red), *Klebsiella* species (green) and *Pseudomonas aeruginosa* (blue) according to EUCAST. The dark grey solid vertical line represents Enterobacterales breakpoints for each drug to guide empiric treatment.

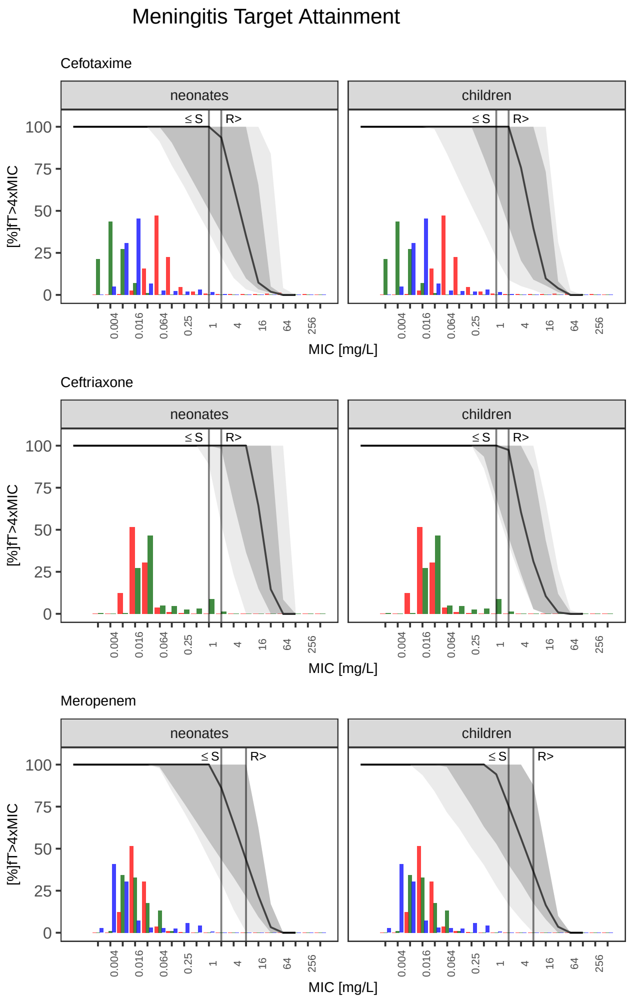

Figure S7: Probability of Target Attainment (100%fT>4xMIC) for simulated dose regimen in the meningitis subpopulation. Solid line Median for common regimen; dark grey area 90% confidence interval for common regimen; light grey area 5th percentile of min regimen to 95th percentile of max regimen. Colored histograms refer to the MIC distribution for common pathogens *Neisseria meningitidis* (green), *Streptococcus pneumoniae* (blue) and *E. coli* (red) according to EUCAST. The dark grey solid vertical line represents Enterobacterales breakpoints for each drug to guide empiric treatment

.

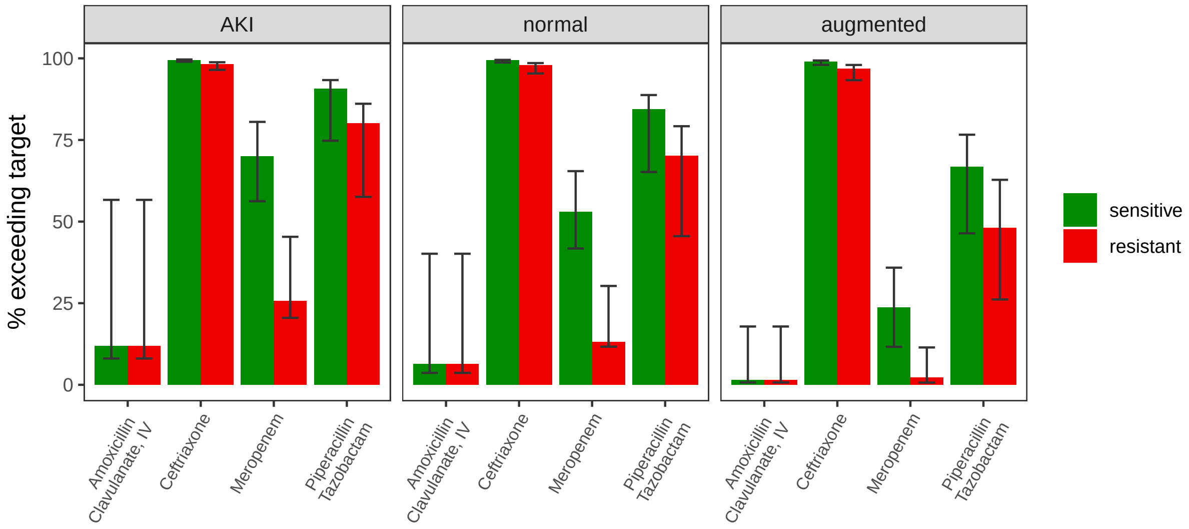

Figure S8: Coverage calculated as percentage of individuals above the PKPD target of 100%fT>MIC to show impact of renal function when treating sepsis. Each panel represents acute kidney injury (AKI), normal renal function and hyperfiltration leading to augmented clearance. group. Each bar represents the coverage of the common regimen, with the error bar showing results of the min to max simulated regimen. Bars are split by sensitive and resistant breakpoints

.
