# Supplementary material for: Variation in Target Attainment of Beta‐Lactam Antibiotic Dosing Between International Pediatric Formularies
Source: Clin Pharmacol Ther. 2021 Feb 28;109(4):958–70. doi: 10.1002/cpt.2180 (PMC8358626; doi:10.1002/cpt.2180)
Supplement: Supplementary file 2 — Table S1 [file CPT-109-958-s004.docx]

|  | | | BNF for Children | Blue Book | Red Book | WHO Pocket-Book | NCDC | SPC | Nelson AAP | DGPI Hand-book | Swiss PedDose | Kinder-formularium NL/DE | common | min | max |
| --- | --- | --- | --- | --- | --- | --- | --- | --- | --- | --- | --- | --- | --- | --- | --- |
|  |  |  | Pneumonia |  |  |  |  |  |  |  |  |  |  |  |  |
| Amoxicillin / Clavulanic acid (Amox content) | Neonates | IV | 50 mg/kg/d, q12h | **0-28d**  60 mg/kg/d, q12h | 90 mg/kg/d, q12h | - | 40 mg/kg/d, q12h | **<3m <4kg**  50mg/kg/d q12h |  | 60-100 mg/kg/d q8h | **<8d**  100 mg/kg/d q12h  **8-28d**  150 mg/kg/d q8h | **<7d, <2kg**  50mg/kg/d q12h  **<7d >=2kg**  75mg/kg/d q8h  **7d -28d** **<2kg**  75mg/kg/d q8h  **7d -28d >=2kg**  100mg/kg/d q6h | 50 mg/kg/d, q12h | 40 mg/kg/d, q12h | 100 mg/kg/d q8h |
|  |  | p.o.  4:1 Susp. | **<1m**  18.75mg/kg/d, q8h | **<28d**  18.75mg/kg/d, q8h  **1m-3m**  18.75-37.5mg/kg/d, q8h |  |  |  | - | **>2kg**  30 mg/kg/d q12h | 50-80 mg/kg/d q8h |  |  | 37.5 mg/kg/d q8h | 18.75 mg/kg/d q8h | 80 mg/kg/d q8h |
|  | Infants/ Children | IV | **1 – 2m**  50mg/kg/d q12h  **3m – 12yrs**  75 mg/kg/d, q8h | **1m-3m**  90 mg/kg/d, q8h  **3m – 18yrs**  90-120 mg/kg/d, q6-8h | - | - |  | **<3m <4kg**  50mg/kg/d q12h  **>3m**, **<40kg**  75mg/kg/d q8h  **>40kg**  3g q8h |  | **<1 yrs**  60-100 mg/kg/d q8h  **1-12 yrs**  60-100 mg/kg/d q8h | **>1m**  99-200 mg/kg/d  q6-8h, max 200mg/kg/d | **<40kg**  100 mg/kg/d q6-8h  **>=40kg**  2-4g/d q8-12h | **1 – 2m** 50mg/kg/d q12h  **3m – 12yrs**  75 mg/kg/d, q8h | **<40kg**  75mg/kg/d q8h  **>40kg**  3g/d q8h | 200 mg/kg/d, q6h  max 8g/d |
|  |  | p.o.  4:1 Susp | **1m – 5yrs**  37.5mg/kg/d q8h  **6 – 11yrs**: 45mg/kg/d q8h | 18.75-37.5mg/kg/d q8h |  |  |  | **<40kg**  20-60 mg/kg/d q8h | **29-60d** 30mg/kg/d q12h  **≥3m**  20-40 mg/kg/d q8h  max 1.5 g/d | 50-80 mg/kg/d q8h | **>1 m**  80 mg/kg/d q12h, max 2.625g/d | **<40kg**  60-80 mg/kg/d q8h  **≥40kg**  2g/d q12h, max 3g/d q8h | **1m – 5yrs**  37.5mg/kg/d q8h  **6 – 11yrs**: 45mg/kg/d q8h | 18.75 mg/kg/d q8h | **<40kg**  80 mg/kg/d q8h  **≥40kg**  3g/d q8h |
|  | Adolescents | IV | **>12yrs**  75 mg/kg/d, q8h  max 3g/d | 90-120 mg/kg/d, q6-8h  max 1.2g/dose | - | - |  | **<40kg**  75mg/kg/d q8h  **>40kg**  3g/d q8h |  | **>12yrs**  3.6-6.6g/d q8h  max 6.6g/d | - | **<40kg**  100 mg/kg/d q6-8h  **≥40kg**  2-4g/d q8-12h | **>12yrs**  75 mg/kg/d, q8h  max 3g/d q8h | **<40kg**  75mg/kg/d q8h  **≥40kg**  2g/d q12h | 200 mg/kg/d, q6h  max 6g/d |
|  |  | p.o.  4:1 Susp | 1500mg/d, q8h |  |  |  |  | **≥40kg**  1.5g/d q8h | 20-40 mg/kg/d q8h  max 1.5 g/d | 1.5g/d q8h  max 3.75g AmoxClav | - | **<40kg**  60-80 mg/kg/d q8h  **≥40kg**  2g/d q12h, max 3g/d | 1500mg/d, q8h | **>40kg**  1.5g/d q8h | **<40kg**  80 mg/kg/d q8h  **≥40kg**  3g/d q8h |
| Benzylpenicillin (Penicillin G, Na-salt) | Neonates | IV | **<7d**  50-75 mg/kg/d, q8-12h  **7d–28d**  75 mg/kg/d, q8h | **<7d**  50 mg/kg/d, q12h  **7d–28d**  75-150 mg/kg/d, q8h | **≤7d**  100k (60mg) IU/kg/d, q12h  **7-28d**  150k (90mg) IU/kg/d, q8h | **≤7d**  100k  (60mg) IU/kg/d, q12h  **8d-2m**  200k IU/kg/d, q6h | 200k-400k U/kg/d q6h | **<1w**  50 mg/kg/d q12h  **1-4wks**  75 mg/kg/d q8h | **≤28 d**  **≤7d** 100k IU/d q12h  **8-28d** 150k IU/d q8h | - |  | **<7d, <2kg**  50k IU kg/d q12h  **<7d ≥2kg**  75k IU/kg/d q8h  **7d -28d** **<2kg**  75k IU /kg/d q8h  **7d -28d** ≥2kg  100k IU/kg/d q6h | 90mg/kg/d q8h | **≤7d**  50k (30mg) IU/kg/d, q12h  **7-28d**  75mg/kg/d, q8h  **>28do**  100k (60mg) IU/kg/d, q6h | **≤7d**  90 mg/kg/d, q8h  **7d–28d**  150 mg/kg/d, q8h  **>1m**  300mg/kg/d, q4h |
|  | Infants/ Children | IV | 100-300 mg/kg/d, q4-6h  max. 2.4g/dose q4h | 100 -300mg/kg/d, q4-6h | 100k-300k IU/kg/d, q4-6h max 24M | 200k IU/kg/d, q6h |  | **>1m**  100mg/kg/d q6h, max 4g/d | **29d -60d**  200k IU/d q6h  **>60d**  100k– 300k IU/kg/d q4-6h, max 24mio IU | **3mo-12yrs**  100k – 500k U/kg/d, q4-6h | 0.2 M IU/kg/d  q6h, max 8M IU/d | 100k IU – 400k IU/kg/d q4-6h, max 24M IU/d | 100 mg/kg/d, q6h  max 4g | 100k (60mg) IU/kg/d, q6h  Max 24M (14.4g/d) | 300mg/kg/d, q4h  2.4g/dose q4h |
|  | Adolescents | IV |  |  |  |  |  | **>12yrs**  600mg – 3.6g q4-6h |  | **>12yrs**  1-3mio U/d q4-6h |  |  |  |  |  |
| Ampicillin/ Sulbactam (Ampicillin content) | Neonates | IV | - | - | - | - | - | **<14d**  50mg/kg/d, q12h  **≥14d**  100mg/kg/d q6-8h | - | - | - | **<7d**  50mg/kg/d q12h  **≥7d**  100mg/kg/d q6-8h | **<14d**  50mg/kg/d, q12h  **≥14d**  100mg/kg/d q6h | **<14d**  50mg/kg/d, q12h  **≥14d**  100mg/kg/d q6h | **<14d**  50mg/kg/d, q12h  **≥14d**  100mg/kg/d q6h |
|  | Infants/ Children | IV | - | - | - | - | - | 100mg/kg/d q6-8h, max TDD 8g | **<40kg**  200mg/kg/d q6h  **>40kg**  4-8g/d q6h | **3m-1yr**  100mg/kg/d q8h  **1yr-12yrs**  100-150mg/kg/d q8h | - | 100mg/kg/d q6-8h | 100mg/kg/d q6h | 100mg/kg/d q6h | **<40kg**  200mg/kg/d q6h  **≥40kg**  12g/d q6h |
|  | Adolescent | IV | - | - | - | - | - | 1.5 – 8g/d, q6-8h max 8g |  | **>12yrs**  2.25-6.75 g, max 12g | - |  | 8g/d q6h | 1.5g/d q8h | **<60kg**  200mg/kg/d q6h  **≥60kg**  12g/d q6h |
|  |  | | Sepsis |  |  |  |  |  |  |  |  |  |  |  |  |
| Amoxicillin / Clavulanic acid (Amox content) | Neonates | | 50 mg/kg/d, q12h | **<28d**  60 mg/kg/d, q12h | - | - | 40 mg/kg/d, q12h | **<3m <4kg**  50mg/kg/d, q12h | - | **<1 yrs**  60-100 mg/kg/d q8h | **<8d**  100mg/kg/d  q12h  **8-28d**  150mg/kg/d  q8h | **<7d, <2kg**  50mg/kg/d q12h  **<7d >=2kg**  75mg/kg/d q8h  **7d -28d** **<2kg**  75mg/kg/d q8h  **7d -28d >=2kg** 100mg/kg/d q6h | 60 mg/kg/d, q12h | 40mg/kg/d q12h | 100 mg/kg/d q8h |
|  | Infants/ Children | | **<3m**  50 mg/kg/d, q12h  **>3m**  75 mg/kg/d, q8h  max 3g/d | **1m-3m**  90 mg/kg/d, q8h  **3m – 12yrs**  90-120 mg/kg/d, q6-8h |  |  |  | **<3m <4kg**  50mg/kg/d, q12h  **>3m, <40kg**  75mg/kg/d q8h  **>40kg**  3g q8h |  | 60-100 mg/kg/d q8h | 150-200mg/kg/d q6-8h, max 8g/d | **<40kg**  100 mg/kg/d q6-8h  **≥40kg**  2-4g/d q8-12h | **<3m**  60 mg/kg/d, q12h  **>3m – 12yrs**  90 mg/kg/d, q8h  max 1.2g/dose | **<3m <4kg**  50mg/kg/d, q12h  **>3m <40kg**  75mg/kg/d q8h  **>40kg**  3g q8h | 100 mg/kg/d |
|  | Adolescents | |  | **>12yrs**  75-100 mg/kg/d q6-8h, max 1.2g/dose |  |  |  |  |  | **>12yrs**  3.6-6.6g/d q8h  max 6.6g/d |  |  |  |  | 6.6g/d q8h |
| Cefotaxime | Neonates | | **<7d**  100 mg/kg/d, q12h  **7d–20d**  150 mg/kg/d, q8h  **21d-28d**  150-200 mg/kg/d, q6-8h | **<7d**  50-100 mg/kg/d, q12h  **7d–21d**  75-150 mg/kg/d, q8h  **21d-28d**  100-200 mg/kg/d, q6h | **≤7d (GA≥32w)**  50 mg/kg/d, q12h  **>7d (GA≥32w)**  50 mg/kg/d, q8h  **<14d (GA<32w)**  50 mg/kg/d, q12h  **≥14d (GA<32w)**  50mg/kg/d, q8h | **<7d**  150 mg/kg/d, q8h  **2w-4w**  200 mg/kg/d, q6h | 100 mg/kg/d, q6-8h | **≤7d**  100 mg/kg/d, q12h  **8d -1m**  150 mg/kg/d, q8h | **≤2kg**  **≤7d** 100mg/kg/d q12h  **8-28d** 150 mg/kg/d q8h  **>2kg**  **≤7d** 100 mg/kg/d q12h  **8-28d** 150mg/kg/d q6h | **<3m**  100-200mg/kg/d q8-12h | - | **<7d**  100 mg/kg/d q12h  **7d -28d**  150mg/kg/d q8h,  max 200mg/kg/d q6h | **≤7d**  100 mg/kg/d, q12h  **>7d**  150mg/kg/d q8h | **<7d**  50 mg/kg/d, q12h  **7d–20d**  75 mg/kg/d, q8h  **21d-28d**  75-100 mg/kg/d, q6-8h | **<7d**  150 mg/kg/d, q8h  **2w-4w**  200 mg/kg/d, q6h |
|  | Infants/ Children | | 200 mg/kg/d, q6h max TDD 12g | 150-200 mg/kg/d, q6-8h  Max TDD 12g | 150-180 mg/kg/d, q8h | **>2m**  200 mg/kg/d, q6h |  | **>1m**  50-150mg/kg/d q6-12h  **>3m- 12yrs**, **<50kg**  50-150mg/kg/d q6-12h  **≥50kg**  6-12g/d, q6-8h, max 12g/d | **29d -60d**  200mg/kg/d q6h  **>60d**  150-180mg/kg/d q8h  max 8g/d | **3mo-1yr**  100-150mg/kg/d q6-12h  **1-12yrs**  100-150mg/kg/d q6-12h | - | 150mg/kg/d q8h, max 12g/d | **≤40kg**  150mg/kg/d q8h  **>40kg**  6g/d q8h | **3m- 12yrs**, **<50kg**  50 mg/kg/d q6h  **≥50kg**  6g/d, q6h, max 12g/d | **>1m**  200 mg/kg/d, q8h  Max 12g/d |
|  | Adolescents | |  |  |  |  |  |  |  | 3-6g/d q6-12h | - |  |  |  |  |
| Ceftazidime | Neonates | | **<7d**  25 mg/kg/d, q24h  **7d–20d**  50 mg/kg/d, q12h  **>21d**  75 mg/kg/d, q8h  double dose in severe infection | **<7d**  25-50 mg/kg/d ÷ q24h  **7d–21d**  50-100 mg/kg/d, q12h  **>21d**  75-150 mg/kg/d, q8h | **≤7d (GA ≥32w)**  50 mg/kg/d, q12h  **>7d (GA ≥32w)**  50 mg/kg/d, q8h  **<14d (GA <32w)**  50 mg/kg/d, q12h  **≥14d (GA <32w)**  50mg/kg/d, q8h | No info | 50-100 mg/kg/d, q12h | **≤2 m**  25-60 mg/kg/d q12h | **≤2kg**  **≤7d** 100mg/kg/d q12h  **8-28d** 150 mg/kg/d q8h  >**2kg**  **≤7d** 100 mg/kg/d q12h  **8-28d** 150mg/kg/d q8h | **<3m**  100-200mg/kg/d q8-12h |  | **<7d, <2kg**  50mg/kg/d q12h  **<7d >=2kg**  100mg/kg/d q12h  **7d -28d** **<2kg**  100mg/kg/d q12h  **7d -28d >=2kg**  150mg/kg/d q8h | **GA <32w**  **<14d** 50mg/kg/d, q12h  **≥14d** 50mg/kg/d, q8h  **GA ≥32w**  **≤7d** 50 mg/kg/d, q12h  **>7d** 50mg/kg/d, q8h  **>28d**  100 mg/kg/d, q8h | **<7d**  25 mg/kg/d, q24h  **7d–20d**  50 mg/kg/d, q12h  **>21d**  75 mg/kg/d, q8h | **≤2kg**  **≤7d** 100mg/kg/d q12h  **8-28d** 150 mg/kg/d q8h  **>2kg**  **≤7d** 100 mg/kg/d q12h  **8-28d** 150mg/kg/d q8h |
|  | Infants/ Children | | **>1m**  75 mg/kg/d, q8h  max TDD 6g | **>1m**  75-150 mg/kg/d, q8h  max 6g/d | 90-150 mg/kg/d, q8h  200-300 mg/kg/d, q8h (severe Pseudomonas infections) |  |  | **<40kg** **>2m**  150 mg/kg/d q8h  **≥40** kg  6g/d q8h  max 6g/d | **29d -60d**  150mg/kg/d q8h  **>60d**  90–150 mg/kg/d q8h, max 6 g/d  Pseudomonas 200–300 mg/kg/d max 12 g/d | **3m-1yr**  100-150mg/kg/d q8-12h  **1-12yrs**  100-150mg/kg/d q8-12h | 150-200mg/kg/d q6-8h, max 6g/d | 100-150 mg/kg/d q8h, max 6g/d | **>3m**  150 mg/kg/d, q8h  max 6g/d | 75 mg/kg/d, q8h  max 6g/d | **29d -60d**  150mg/kg/d q8h  **>60d**  300 mg/kg/d, q8h, max 12 g/d |
|  | Adolescents | |  |  |  |  |  |  |  | **>12yrs**  2-6g/d q8-12h |  |  |  |  |  |
| Ceftriaxone | Neonates | | **<15d**  20-50 mg/kg/d, q24h  **15d-28d**  50-80 mg/kg/d, q24h | **0-28d**  25-50 mg/kg/d, q24h | **≤28d** 50mg/kg/d, q24h | **<2m**  100 mg/kg/d, q12-24h | 50-75mg/kg/d, q12h | **>41wks PMA**  **<14d**  20-50 mg/kg/d, q24h  **≥14d**  50-100mg/kg/d, q24h | **<60d >2kg**  50mg/kg/d, q24h | **<60d**  100-200mg/kg/d q8-12h |  |  | **≤7d**  50 mg/kg/d, q12h  **>7d**  75 mg/kg/d q24h | **<15d**  20 mg/kg/d, q24h  **<28d**  25 mg/kg/d, q24h  **≥28d**  50mg/kg/d, q24h | **<60d**  200mg/kg/d q8h |
|  | Infants/ Children | | **<50kg**  50-80 mg/kg/d, q24h max 4g/d  **≥50kg >9yr**  1000-2000mg/d, q24h | 50-80 mg/kg/d, q24h  max 4g/d | >28d  50-75 mg/kg/d, q24h  100 mg/kg/d, q12-24h (severe) | <2m  100 mg/kg/d, q12-24h  >2m  80 mg/kg/d, q24h |  | <50kg  50-100mg/kg/d, q24h, max 4g  >50kg  2g q24h | 50–75 mg/kg/d q24h, max 2 g/d | 3mo-1yr  50-75mg/kg/d q12-24h  1-12yrs  50-75mg/kg/d q12-24h | >=1m  100 mg/kg/d  q24h, max 2g/d | >=1m  100 mg/kg/d q24h, max 4g/d | 75 mg/kg/d q24h ≥50kg  1500 mg/d, q24h | 50 mg/kg/d q24h | 100 mg/kg/d q24h |
|  | Adolescents | |  |  |  |  |  |  |  | >12yrs  1-4g/d q12-24h, max 4g |  |  | 1500 mg/d, q24h | 1000 mg/d q24h | 100 mg/kg/d q24h, max 4g |
| Meropenem | Neonates | | **<7d**  40 mg/kg/d, q12h  **7d–28d**  60 mg/kg/d, q8h | **<7d**  80 mg/kg/d, q12h  **7d–28d**  120 mg/kg/d, q8h | **GA <32w**  **<14d**  40 mg/kg/d, q12h  **≥14d**  60 mg/kg/d, q8h  **GA ≥32w**  **<14d**  60 mg/kg/d, q8h  **≥14d**  90 mg/kg/d, q8h | - | - | - | **≤2kg**  **≤7d** 40mg/kg/d q12h  **8-28d** 60 mg/kg/d q8h  **>2kg**  **≤7d** 60mg/kg/d q8h  **8-28d** 90mg/kg/d q8h | **Preterm <2kg**  40 mg/kg/d q12h  **Neonates <3mo**  60-80 mg/kg/d q8h | **GA <32w, 14d PNA**  40mg/kg/d q12h  **GA <32w, ≥14d PNA**  60mg/kg/d q8h  **GA 32w – 14d, <14d PNA**  60mg/kg/d q8h  **GA 32w – 28d, ≥14d**  **PNA**  90mg/kg/d q8h | **<7d**  40 mg/kg/d q12h  **7-28d**  60 mg/kg/d q8h | **Preterm**  **<2kg**  40 mg/kg/d q12h  **Neonates <3mo**  60 mg/kg/d q8h | **<7d**  40 mg/kg/d q12h  **7d-28d**  60 mg/kg/d q8h | **≤7d**  80 mg/kg/d, q12h  **7d–28d**  120 mg/kg/d, q8h  **>1m**  120 mg/kg/d, q8h  Max 1.5g/d |
|  | Infants/ Children | | **>1m**  **<50kg** 30-60 mg/kg/d, q8h  **≥50kg** 1500-3000 mg/d, q8h | **>1m**  30-120 mg/kg/d, q8h  Max 1.5g | 60 mg/kg/d, q8h |  | 60 mg/kg/d, q8h | **<50kg >3m**  30-60mg/kg/d, q8h  **>50kg**  1.5-3g/d, q8h | **29d -60d**  90mg/kg/d q8h  **>3m**  60 mg/kg/d, q8h, max 3 g/d | **3m-12yrs**  60 mg/kg/d q8h | 60mg/kg/d q8h, max 3g/d | 60 mg/kg/d q8h, max 6g/d | **3m-12yrs**  60 mg/kg/d q8h | **<50kg**  30mg/kg/d, q8h  **>50kg**  1.5g/d, q8h | **<50kg**  120mg/kg/d, q8h  **>50kg**  3g/d, q8h |
|  | Adolescents | |  | - |  |  |  |  |  | **>12yrs**  1.5-3g q8h |  |  | **>12yrs**  3g q8h |  |  |
| Piperacillin-tazobactam (piperacillin based dose) | Neonates | | **<1mo**  240 mg/kg/d, q8h | **0-28d**  270 mg/kg/d, q8h | **≤28d**  **PMA ≤30w** 300 mg/kg/d, q8h  **PMA >30w** 320mg/kg/d, q6h | - | 200-400 mg/kg/d, q6-8h | - | **≤2kg**  **≤7d** 300mg/kg/d q8h  **8-28d** 320mg/kg/d q6h  **>2kg**  **≤7d** 320mg/kg/d q6h  **8-28d** 320mg/kg/d q6h | **<30 wks GA**  300 mg/kg/d, q8h  **30-35 wks**  320 mg/kg/d, q6h  **>35 wks**  480 mg/kg/d, q4h | **<28d**  300mg/kg/d, q8h |  | **<1mo** 360mg/kg/d q6-12h | 200mg/kg/d, q6h | **<30 wks GA**  300 mg/kg/d, q8h  **30-35 wks**  320 mg/kg/d, q6h  **>35 wks**  480 mg/kg/d, q4h |
|  | Infants/ Children | | **>1m**  240-320 mg/kg/d, q6-8h max 16g/d q6h | 270-360 mg/kg/d, q6-8h  max 13.5g/d | 240-300 mg/kg/d, q6-8h |  |  | **2-12yrs**  320 or 300 mg/kg/d, q6 or 8h | **29d -60d**  320mg/kg/d q6h  ≤4**0kg**  240-300 mg/kg/d, q8h, max TDD 16g | **<40kg**  225 mg/kg/d q6-8h  **>40kg**  13.5g/d q6-8h, max 16g | 300-400 mg/kg/d  q6-8h, max 4g/dose | 320mg/kg/d q6h, max 16g/d | 360 mg/kg/d, q6h | 225 mg/kg/d q8h | 400 mg/kg/d, q6h |
|  | Adolescents | | **>12yrs**  12g/d, q8h |  |  |  |  | **>12yrs**  12g/d q8h |  |  |  |  | 13.5g/d, q8h | 4g/d q6h | 16g/d q6h |
|  | | | Meningitis |  |  |  |  |  |  |  |  |  |  |  |  |
| Cefotaxime | Neonates | | **<7d**  100 mg/kg/d, q12h  **7d–20d**  150 mg/kg/d, q8h  **21-28d**  150-200 mg/kg/d, q6-8h | **<7d**  50-100 mg/kg/d, q12h  **7d–21d**  75-150 mg/kg/d, q8h  **21d-28d**  100-200 mg/kg/d, q6h | **≤7d (GA ≥32w)**  50 mg/kg/d, q12h  **>7d (GA ≥32w)**  50 mg/kg/d, q8h  **<14d (GA <32w)**  50mg/kg/d, q12h  **≥14d (GA <32w)**  50mg/kg/d, q8h | **<7d**  100 mg/kg/d, q12h  **>7d-14d**  150 mg/kg/d, q8h  **>14d**  200 mg/kg/d, q6h | 200 mg/kg/d, q6h | **≤7d**  100 mg/kg/d, q12h  **8d -1m**  150 mg/kg/d, q8h | **≤28 d, ≤2kg ≤7d** 100mg/kg/d q12h  **8-28d** 150 mg/kg/d q8h  **≤28 d, >2kg ≤7d** 100 mg/kg/d q12h  **8-28d** 150mg/kg/d q6h | 100-200mg/kg/d q8-12h | - | **<7d**  100 mg/kg/d q12h  **7d -28d**  150mg/kg/d q8h, max 200mg/kg/d q6h | **<7d**  100 mg/kg/d, q12h  **7d–28d**  150 mg/kg/d, q8h  **>28d**  200mg/kg/d, q6h | **<7d**  50mg/kg/d, q12h  **7d–20d**  75mg/kg/d, q8h  **21d-28d**  100mg/kg/d, q6h | 200 mg/kg/d, q6h |
|  | Infants/ Children | | **>1m**  200mg/kg/d, q6h  Max 12g/d | **>1m**  150-200 mg/kg/d, q6-8h  Max 12g/d | **>1m**  225-300 mg/kg/d, q8h  Max 2g/dose | **>1m**  200 mg/kg/d, q6h |  | **<50kg**  150-200mg/kg/d q6-8h  **≥50kg**  6-12g/d, q6-8h, max 12g/d | **29d -60d**  200mg/kg/d q6h  **>60d**  200-225mg/kg/d q8h  max 12g/d | **1m-3m**  100-200mg/kg/d q8-12h  **>3mo**  200mg/kg/d q6-8h | - | 150mg/kg/d q8h, max 12g/d | 200mg/kg/d, q6h  Max 12g/d | 150mg/kg/d, q8h  Max 12g/d | 225mg/kg/d, q6h  Max 12g/d |
|  | Adolescents | |  |  |  |  |  |  |  | **>12yrs**  12g/d q6-8h | - |  |  |  |  |
| Ceftriaxone | Neonates | | **≤15d**  50 mg/kg/d, q24h  **>15d**  80 mg/kg/d, q24h max 4g/d | **<28d**  25-50 mg/kg/d, q24h |  | **<7d** 100mg/kg/d q12h  **>7d** 150mg/kg/d q12h | 100 mg/kg/d, q12h | **>41wks PMA <14d**  50 mg/kg/d, q24h  **≥14d**  80-100mg/kg/d, q24h | **>2kg**  50mg/kg/d, q24h | **<60d**  100-200mg/kg/d q8-12h |  |  | **≤7d**  100 mg/kg/d, q12h  **>7d**  75 mg/kg/d, q24h | **<28d**  25 mg/kg/d, q24h  **≥28d**  80 mg/kg/d, q24h | **<60d**  200mg/kg/d q8h |
|  | Infants/ Children | | **<50kg**  80 mg/kg/d, q24h, max 4g/d  **≥50kg >9yrs**  2000-4000 mg/d, q24h | 50-80 mg/kg/d, q24h  max TDD 4g | **>28d**  50-75 mg/kg/d, q24h  100 mg/kg/d, q12-24h (severe) | 100 mg/kg/d, q12-24h |  | **<50kg**  80-100mg/kg/d, q24h, max 4g  **≥50kg**  2-4g q24h | **29-60d**  50mg/kg/d q24h  **>60d**  100 mg/kg/d q12h, max 4 g/d | **>3mo**  100mg/kg/d q24h | 100 mg/kg/d q24h, max 4g/d | 100 mg/kg/d q24h, max 4g/d | 75 mg/kg/d, q24h  >40kg  3g/d q24h | **<50kg**  80mg/kg/d, q24h, max 4g  **≥50kg**  2g q24h | **<50kg**  100mg/kg/d, q24h, max 4g  **≥50kg**  4g q24h |
|  | Adolescents | | **≥12yrs**  2000-4000 mg/d, q24h | 80 mg/kg/d, q24h  max TDD 4g |  |  |  |  |  | **>12yrs**  1-2g/d q12-24h, max 4g |  |  |  |  |  |
| Meropenem | Neonates | | **<7d**  80 mg/kg/d, q12h  **>7d**  120 mg/kg/d, q8h | **≤7d**  80 mg/kg/d, q12h  **7d–28d**  120 mg/kg/d, q8h | **GA <32w**  **<14d** :20 mg/kg/d, q12h  **≥14d** 20 mg/kg/d, q8h  **GA ≥32w**  **<14d** 20 mg/kg/d, q8h  **≥14d** 30 mg/kg/d, q8h | - | - | - | **≤2kg**  **≤7d** 80mg/kg/d q12h  **8-28d** 120 mg/kg/d q8h  **>2kg**  **≤7d** 120mg/kg/d q8h  **8-28d** 120mg/kg/d q8h | **Preterm <2kg**  40 mg/kg/d q12h  **Neonates <3mo**  80 mg/kg/d q8h | **<32w GA, preterm**  80mg/kg/d q12h  **<32w GA, term**  120mg/kg/d q8h  **≥32w GA – 28d**  120mg/kg/d q8h | **<7d PNA**  40mg/kg/d q12h  **7d – 28d**  60 mg/kg/d q8h | **<7d**  80 mg/kg/d, q12h  **>7d**  120 mg/kg/d, q8h | **Preterm <2kg**  40 mg/kg/d q12h  **Neonates <3mo**  30 mg/kg/d q8h | **≤7d**  80 mg/kg/d, q12h  **>7d**  120 mg/kg/d, q8h |
|  | Infants/ Children | | **<50kg**  120 mg/kg/d, q8h  **≥50kg**  6000 mg/d, q8h | **>1m**  30-120 mg/kg/d, q8h  Max 1.5g | 120 mg/kg/d, q8h |  | 75 mg/kg/d, q8h | **<50kg >3m**  120mg/kg/d, q8h  **>50kg**  6g/d, q8h | **29d -60d**  120mg/kg/d q8h  **>3m**  120 mg/kg/d, q8h, max 6 g/d | **3m-12yrs**  80-120mg/kg/d q8h | **>1m**  120mg/kg/d q8h, max 6g/d | **≥1m**  120mg/kg/d q8h max 6g/d | **>3m**  **<50kg** 120 mg/kg/d, q8h  **≥50kg** 6000 mg/d, q8h | 60 mg/kg/d, q8h | **<50kg** 120 mg/kg/d, q8h  **≥50kg** 6000 mg/d, q8h |
|  | Adolescents | |  |  |  |  |  |  |  | **>12yrs**  6g q8h |  |  |  |  |  |

**Table S1: Dosing guidance for beta-lactam antibiotics listed in the WHO Essential Medicines List for Children (EMLc). Dosing recommendations were extracted and calculated to total daily doses (mg/kg/d) where appropriate. d = ds old, m = months old, yrs = years old, q4h = every 4 hours, q6h = every 6 hours, q8h = every 8 hours, q12h = every 12 hours, q24h = every 24 hours, q48h = every 48 hours, GA = gestational age, PMA = post menstrual age, IU = international unit. Shaded cells show syndrome specific dose recommendations.**
