# Supplementary material for: Variation in Target Attainment of Beta‐Lactam Antibiotic Dosing Between International Pediatric Formularies
Source: Clin Pharmacol Ther. 2021 Feb 28;109(4):958–70. doi: 10.1002/cpt.2180 (PMC8358626; doi:10.1002/cpt.2180)
Supplement: Supplementary file 3 — Table S2 [file CPT-109-958-s001.docx]

**Table S2:** Maximum MICs attained by minimum, common and maximum dose regimens for each example pathogen

| **Syndrome** | **Drug** | **age group** | **min** | | **common** | | **max** | | **ECOFF** | | | **Empiric**  **target MIC** |
| --- | --- | --- | --- | --- | --- | --- | --- | --- | --- | --- | --- | --- |
| **Pneumonia** |  |  | **MIC** | **4xMIC** | **MIC** | **4xMIC** | **MIC** | **4xMIC** | ***Staph. aureus*** | ***Step. pneumoniae*** | ***H. influenzae*** |  |
|  | Ampicillin | neonate | 0.064 | 0.016 | 0.064 | 0.016 | 0.064 | 0.016 | 8 (CLSI) | 0.064 | 1 | 2 |
|  |  | child | 0.25 | 0.032 | 0.5 | 0.125 | 1 | 0.25 |  |  |  |  |
|  | Co-Amoxicillin -IV | neonate | 1 | 0.25 | 1 | 0.25 | 4 | 1 | 4 (CLSI) | 0.064 | 2 |  |
|  |  | child | 0.5 | 0.125 | 1 | 0.25 | 2 | 0.5 |  |  |  |  |
|  | Co-Amoxicillin -oral | neonate | 1 | 0.25 | 2 | 0.5 | 4 | 1 | 4 (CLSI) | 0.064 | 2 | 2 |
|  |  | child | 0.5 | 0.125 | 1 | 0.25 | 2 | 0.5 |  |  |  |  |
|  | Benzylpenicillin | neonate | 0.016 | 0.004 | 0.125 | 0.016 | 0.125 | 0.016 | 0.125 | 0.064 | 1 | 0.25 |
|  |  | child | 0.032 | 0.008 | 0.064 | 0.016 | 1 | 0.25 |  |  |  |  |
| **Sepsis** |  |  |  |  |  |  |  |  | ***P. aeruginosa*** | ***E. coli*** | ***K. pneumoniae*** |  |
|  | Cefotaxime | neonate | 2 | 0.5 | 4 | 1 | 8 | 2 | 32 | 0.25 | 0.25 | 1 |
|  |  | child | 2 | 0.5 | 2 | 0.5 | 8 | 2 |  |  |  |  |
|  | Ceftazidime | neonate | 2 | 0.5 | 4 | 1 | 16 | 4 |  |  |  |  |
|  |  | child | 4 | 1 | 4 | 1 | 8 | 2 |  |  |  |  |
|  | Ceftriaxone | neonate | 8 | 2 | 16 | 4 | 32 | 8 | ND | 0.125 | 0.125 | 1 |
|  |  | child | 8 | 2 | 8 | 2 | 8 | 2 |  |  |  |  |
|  | Co-Amoxicillin -IV | neonate | 1 | 0.25 | 1 | 0.25 | 4 | 1 | ND | ND | ND | 2 |
|  |  | child | 0.5 | 0.125 | 0.5 | 0.125 | 4 | 1 |  |  |  |  |
|  | Meropenem | neonate | 2 | 0.5 | 4 | 1 | 16 | 4 | 2 | 0.125 | 0.125 | 2 |
|  |  | child | 4 | 1 | 4 | 1 | 8 | 2 |  |  |  |  |
|  | Piperacillin/Tazobactam | neonate | 16 | 4 | 32 | 8 | 64 | 16 | 16 | 8 | 8 | 4 |
|  |  | child | 4 | 1 | 8 | 2 | 16 | 4 |  |  |  |  |
| **Meningitis** |  |  |  |  |  |  |  |  | ***N. meningitidis*** | ***E. coli*** | ***Strep. pneum*** |  |
|  | Cefotaxime | neonate | 2 | 0.5 | 4 | 1 | 16 | 4 | 0.016 | 0.25 | 0.064 | 1 |
|  |  | child | 1 | 0.25 | 8 | 2 | 8 | 2 |  |  |  |  |
|  | Ceftriaxone | neonate | 8 | 2 | 32 | 8 | 128 | 32 | ND | 0.125 | 0.064 | 1 |
|  |  | child | 4 | 1 | 4 | 1 | 8 | 2 |  |  |  |  |
|  | Meropenem | neonate | 2 | 0.5 | 4 | 1 | 16 | 4 | ND | 0.125 | 0.016 | 2 |
|  |  | child | 1 | 0.25 | 8 | 2 | 8 | 2 |  |  |  |  |
