# Supplementary material for: Variation in Target Attainment of Beta‐Lactam Antibiotic Dosing Between International Pediatric Formularies
Source: Clin Pharmacol Ther. 2021 Feb 28;109(4):958–70. doi: 10.1002/cpt.2180 (PMC8358626; doi:10.1002/cpt.2180)
Supplement: Supplementary file 4 — Table S3 [file CPT-109-958-s003.docx]

**Table S3:** Literature models used in target attainment simulations.

|  | Drug | model type | covariates | n | age range | indication |
| --- | --- | --- | --- | --- | --- | --- |
| Lonsdale et al. | Benzylpenicillin  Co-amoxiclav (IV)  Piperacillin-Tazobactam  Cefotaxime  Meropenem | joint beta-lactam model, 2cmt, linear elimination | creatinine  PMA  PNA  weight | meta-analysis  6-54 studies per drug with 12-15 patients each | 25 weeks to 82 years | Neonatal, pediatric and adult intensive care |
| deVelde et al. | Co-amoxiclav (oral) | 2cmt. Linear elimination  transit compartment with saturable absorption rate | allometry and maturation function added | 28 | 33+-7 years | healthy male volunteers |
| Soto et al. | Ampicillin | 2cmt, linear elimination | weight  creatinine-clearance  allometry and maturation function added | 47 | 28-85 years | CAP |
| Standing et al. | Ceftriaxone | 3cmt, linear elimination, saturable protein binding | albumin  weight  creatinine  PMA | 81 | 2-45 months | severe acute malnutrition |
| Li et al. | Ceftazidime | 2cmt, linear elimination | creatinine-clearance  allometry and maturation function added | 1975 | 18-89 years | healthy volunteers and patients |
